# Application of Genomics Tools in Wheat Breeding to Attain Durable Rust Resistance

**DOI:** 10.3389/fpls.2020.567147

**Published:** 2020-09-11

**Authors:** Prashanth Babu, Deepak Kumar Baranwal, Harikrishna, Dharam Pal, Hemlata Bharti, Priyanka Joshi, Brindha Thiyagarajan, Kiran B. Gaikwad, Subhash Chander Bhardwaj, Gyanendra Pratap Singh, Anupam Singh

**Affiliations:** ^1^ Indian Agricultural Research Institute (ICAR), New Delhi, India; ^2^ Plant Breeding Institute, The University of Sydney, Cobbitty, NSW, Australia; ^3^ Directorate of Medicinal and Aromatic Plants Research (ICAR), Anand, India; ^4^ Department of Plant Pathology, Washington State University, Pullman, WA, United States; ^5^ AC & RI, Tamil Nadu Agricultural University, Coimbatore, India; ^6^ Indian Institute of Wheat and Barley Research (ICAR), Karnal, India; ^7^ DCM SHRIRAM-Bioseed Research India, ICRISAT, Hyderabad, India

**Keywords:** wheat, rust, resistance, discovery, deployment, genomics, Kompetitive allele-specific PCR assay

## Abstract

Wheat is an important source of dietary protein and calories for the majority of the world’s population. It is one of the largest grown cereal in the world occupying over 215 M ha. Wheat production globally is challenged by biotic stresses such as pests and diseases. Of the 50 diseases of wheat that are of economic importance, the three rust diseases are the most ubiquitous causing significant yield losses in the majority of wheat production environments. Under severe epidemics they can lead to food insecurity threats amid the continuous evolution of new races of the pathogens, shifts in population dynamics and their virulence patterns, thereby rendering several effective resistance genes deployed in wheat breeding programs vulnerable. This emphasizes the need to identify, characterize, and deploy effective rust-resistant genes from diverse sources into pre-breeding lines and future wheat varieties. The use of genetic resistance has been marked as eco-friendly and to curb the further evolution of rust pathogens. Deployment of multiple rust resistance genes including major and minor genes in wheat lines could enhance the durability of resistance thereby reducing pathogen evolution. Advances in next-generation sequencing (NGS) platforms and associated bioinformatics tools have revolutionized wheat genomics. The sequence alignment of the wheat genome is the most important landmark which will enable genomics to identify marker-trait associations, candidate genes and enhanced breeding values in genomic selection (GS) studies. High throughput genotyping platforms have demonstrated their role in the estimation of genetic diversity, construction of the high-density genetic maps, dissecting polygenic traits, and better understanding their interactions through GWAS (genome-wide association studies) and QTL mapping, and isolation of R genes. Application of breeder’s friendly KASP assays in the wheat breeding program has expedited the identification and pyramiding of rust resistance alleles/genes in elite lines. The present review covers the evolutionary trends of the rust pathogen and contemporary wheat varieties, and how these research strategies galvanized to control the wheat killer genus *Puccinia*. It will also highlight the outcome and research impact of cost-effective NGS technologies and cloning of rust resistance genes amid the public availability of common and tetraploid wheat reference genomes.

## Introduction

During the evolution of agriculture, intense changes in the biology of a wider range of traits of cereals have been documented (Preece et al., 2017). These changes assisted in the domestication of wheat and its cultivation in the Fertile Crescent, 10,000 years ago (Nevo et al., 2013). Wheat is a segmental allohexaploid (2n = AABBDD = 42) accommodating three inter-related diploid genomes (Marcussen et al., 2014). Around seven million years ago, diploid progenitors of the AA genome (*Triticum* *urartu*) and BB genome (closely related to *Aegilops speltoides*) diverged from a common ancestor (Marcussen et al., 2014), and during evolution, both of these genomes combined through a polyploidization event which led to the evolution of emmer wheat (*T. turgidum*), an allotetraploid (AABB) around half a million years ago (El Baidouri et al., 2017). Around five million years ago, DD genome (*Ae. tauschii*) evolved from these two diploid progenitors through homoploid speciation (Sandve et al., 2015; El Baidouri et al., 2017; Pont and Salse, 2017). Common wheat (AABBDD) evolved 8,000 to 10,000 years ago through hybridization between *T. turgidum* and *Ae. tauschii*. Further, domestication, cultivation, and selection by mankind have refined the agronomical features of common wheat (Kihara, 1944; McFadden and Sears, 1946). Wheat is an extensively cultivated cereal across the globe covering a wide range of altitudes except for lowland tropical regions (Feldman, 1995). As per global grain production statistics, around 734 million tonnes of wheat was produced in the crop year 2018 at 215 million ha around the world which puts wheat second to maize (*Zea mays* L.) in production among the cereal crops (FAOSTAT, 2020). Common wheat covers around 95% of the total wheat acreage, whereas durum wheat and others occupy the remaining 5% (Shewry, 2009). The world population is projected to reach 9.7 billion by 2050 (Elferink and Schierhorn, 2016), and around 70% in additional production is needed to fulfil the global wheat demand.

Among many biotic stresses which hinder wheat production, rust pathogens are considered as most pressing threats with large economical losses worldwide (Ellis et al., 2014). Stem rust and stripe rust can cause 100% loss, whereas leaf rust can result in 50% loss under severe rust epidemics (Bhardwaj et al., 2019). To minimize the loss incurred by the rust pathogens, there are two effective approaches: 1. chemical control *via* fungicidal spray and 2. genetic control *via* breeding for rust resistance (Asad et al., 2012). The International Maize and Wheat Improvement Centre (CIMMYT), Mexico has been rigorously engaged in improving wheat for rust resistance since its inception in the 1940s, and rich dividends up to 27:1 benefit-to-cost ratio have been attributed to the development of resistant cultivars through genetic approach (Kolmer et al., 2009). The worldwide estimated losses caused by wheat rusts were as high as USD 170 million for stripe (Pakistan) (Hussain et al., 1980), AUD 100–200 million for stem (Australia) (McIntosh et al., 1995) 83 and USD 100 million for leaf rust (Pakistan) (Duveiller et al., 2007), and hence the economic value of rust diseases has been a driving factor for research funding. Breeding for rust resistance is an environmentally safe approach adapted by wheat breeders permitting a reduction in the use of pesticides and greater yield stability (Bariana et al., 2013).

Diverse accessions including diploid progenitors, landraces, old cultivars, and synthetic wheat have an inherent ability to restrict the infection induced by the rust pathogens. Such class of resistance is termed as host resistance, and it can be classified into two broad categories, All Stage Resistance (ASR) and Adult Plant Resistance (APR). ASR is active from the seedling to adult plant stages and generally is only effective against specific races of the pathogen. APR is controlled by non-race specific genes with exceptions where some genes provide race-specific resistance such as *Lr13* (Ellis et al., 2014), while *Lr48* confers hypersensitive response (Bansal et al., 2008). These genes express resistance at post seedling stages when adult plants are close to flag leaf or leaf emergence at boot stage. The effect of each APR gene is partial, not enough to cope up high disease pressure; however, combining APR genes provides high levels of resistance (Singh, 1992). The resistance effect of ASR is neutralized due to selection pressure on the pathogen to evolve or mutate to acquire virulence against resistance genes. Such genes when deployed alone result in boom and bust cycles. The successful breeding for rust resistance requires a deep understanding of evolutionary nature of the pathogen, identification of diverse resistance sources, and judicious deployment of resistance genes in combinations (McIntosh et al., 2001).

Development of gene-specific DNA markers is desirable to expedite pyramiding of rust resistance genes in future wheat varieties. Enormous genome size, complexity, repetitive sequences, and extreme level of similarity between homoeologous genome sequences (95–99% in coding regions), and a higher proportion of repetitive elements (over 80%) have posed a challenge to develop robust DNA markers and draft wheat genome sequences (Borrill et al., 2015). In the last decade, polymerase chain reaction-based markers, including Simple Sequence Repeat (SSR), Sequence Tagged Site (STS), and Cleaved Amplified Polymorphic Sequences (CAPS) were largely explored as promising tools for wheat improvement (Somers et al., 2004; Rosewarne et al., 2013). Wheat genomics has been a witness to the advancement in Next-Generation Sequencing technologies (NGS) from whole-Genome Shotgun (WGS) approach (Brenchley et al., 2012), flow-sorting technology (Vrána et al., 2012), chromosome arm-specific sequencing (IWGSC, 2014; Mayer et al., 2014), long-range sequencing *via* the WGS approach (Chapman et al., 2015), and *de novo* whole-genome assembly (Appels et al., 2018). Development of SNP-based breeder-friendly KASP (Kompetitive Allele-Specific PCR) markers is the outcome of these technologies. These cost-effective technologies have been utilized to isolate rust resistance genes *via* positional cloning and consequently the development of perfect markers for effective ASR and APR genes (Nsabiyera et al., 2020). In this review, we attempt to revisit the nature of rust pathogen resistance sources and available genomic tools useful in gene-specific marker development.

## Wheat Rust Diseases and Their Impact on Productivity

Three rust diseases namely leaf rust caused by *Puccinia triticina* Eriks (Pt), stem rust caused by *P. graminis* Pers. f. sp. *tritici* Eriks. & Henn. (Pgt), and stripe rust caused by *P. striiformis* Westend. f. sp. *tritici (*Pst) afflict wheat. Rust fungi are obligate plant parasites which include over 7,000 species having agricultural and economic importance (Aime et al., 2018). The genus *Puccinia* belongs to the Pucciniale order. The genus evolved around 235 million years ago and is characterized by vast genome size ~380Mb (Tavares et al., 2014). Since the evidence of rust spores dating back to 700BC, rust diseases have been a formidable challenge to wheat cultivation. Roman farmers offered dogs, sheep, incense, and wine to the rust god “Robigus” to be propitious (Zadoks, 1985). Individual species of *Puccinia* require a conducive environment regimes for the proliferation of rust inoculum followed by their dispersal *via* wind (Singh and Rajaram, 1993). Stem rust is usually favored under warm and humid (≤30°C) conditions. Leaf rust pathogen also prefers humidity but comparatively lower temperatures (20–24°C) Stripe rust proliferates under cooler temperatures (12–20°C) (Singh et al., 2011).

The *Puccinia* spp. inciting rusts on wheat are obligate parasites having five spore stages *viz.*, spermatia, aeciospores, urediniospores, teliospores, and basidiospores in its complex life cycle (Money, 2016). Wheat is a primary host for rust fungi. *Berberis* species and *Mahonia* species are alternate/secondary hosts of Pgt and Pst, while *Thalictrum*, *Anchusa*, *Isopyrum*, and *Clematis* are the alternate hosts of Pt (Jin et al., 2010; Wang et al., 2015). Teliosori containing teliospores/teleutospores replace urediniosori as wheat crop matures. The thick-walled, bicelled teliospores can resist overwintering on stubbles. During the onset of spring, the teliospores produce a promycelium which bear four uninucleate haploid basidiospores from each cell. These basidiospores infect the *Berberis* leaves to form pycnial cups/spermagonia containing spermatia/pycniospores and receptive hyphae. Transfer of spermatia (sexual gametes) to receptive hyphae between oppositely charged (+ or −) spermatia and receptive hyphae on barberry leaves with the help of insects completes the sexual reproduction. The resultant dikaryotic spores (aeciospores) are formed on the lower side of the *Berberis* leaves. Aeciospores are dispersed through the wind to fall on wheat leaves to penetrate the stomata by the formation of an appressorium (Money, 2016). Further, infectious mycelia transform into haustorium that initiates further infection and spread in wheat without affecting its plasma membrane. After a week of infection on wheat, urediniospores are produced in millions on a susceptible variety. During the crop season, rust pathogens can produce numerous cycles of urediniospore production, its dispersal takes place through wind, and lead to build-up of epidemic over a vast region (Aime et al., 2018). In countries like India, alternate hosts are not functional and urediniospores act as repeating spores. The rusts survive on off-season wheat crop or grasses and become active in the season to cause infection on wheat through urediniospores (Bhardwaj et al., 2019).

Among rust diseases, leaf rust is the most common wheat disease worldwide (Huerta-Espino et al., 2011). Due to its wide adaptation, leaf rust fungi can initiate their infection and spread under mild temperatures and moist conditions. Although, yield losses caused by this disease display temporal and geographical variation; the economic significance of the disease is substantial (Figueroa et al., 2016). Devastating nature of the rust pathogen became more apparent after the susceptible response of a North American wheat variety “Thatcher” against leaf rust pathotypes (Pt; Anderson, 1963). This epidemic devastated wheat crop on a large area and forced researchers and policymakers to consider Pt as an alarming pathogen in the USA, former USSR, and China. Higher rate of reproduction, the ability to travel long-distance, the potential to survive on various host species and diverse environments increased aggressiveness and genetic diversity of the pathogen (Watson, 1981; Walter et al., 2016). In India, severe leaf rust epidemics occurred due to susceptibility of green revolution wheat varieties “Kalyansona” and “Sonalika” and, contemporary cultivars K68 and Pbc 306 in the Northwest region of the country during 1971–73. Over one-million-ton yield losses were estimated (Joshi et al., 1975). Similarly, another leaf rust epidemic was evident due to susceptibility of Mexican mega-wheat variety “Jupateco 73” in Northwestern Mexico. It occupied 75 per cent of the wheat acreage in the southern Sonora region within four years of its release (Dubin and Torres, 1981). Another leaf rust epidemic was reported in the eastern Prairies due to gained additional virulence against *Lr13* and *Lr21* in 1999 (Aboukhaddour et al., 2020).

Significant yield losses in areas with cool and high-altitude climates are commonly inflicted by stripe rust (Roelfs et al., 1992; Singh et al., 2000). In the majority of wheat-growing areas stripe rust have shown regular regional crop losses of up to 0.1–5% and in few exceptions, the losses extend up to 5–25% (Wellings, 2011) besides, severe epidemics due to the breakdown of genes in larger areas may inflict up to 70% yield losses. One-billion USD annual loss is estimated due to the severity and around ninety per cent of the world’s wheat crop is vulnerable to stripe rust (Schwessinger, 2017). Stripe rust infection at early growth stages can cause complete yield losses through a reduction in grain quality, seed vigor and poor germination in susceptible cultivars (Chen, 2005). In Asia, around 46% of yield losses have been due to stripe rust epidemics (Singh et al., 2004a). Tajikistan and Uzbekistan also witnessed widespread epidemics of stripe rust in 2010 (Sobhy Draz, 2019). In India, stripe rust is a serious threat in over ten million hectares of Northern India. Restriction on the cultivation of a mega wheat variety of this region namely PBW343 was imposed due to acquired virulence of *Yr9* during 2008–09. The disease pressure was recorded up to 80%, which necessitated the cultivation of stripe rust-resistant wheat varieties (Aggarwal et al., 2018; Bhardwaj et al., 2019).

Stem rust can cause greater damage when susceptible wheat cultivars are grown over wide geographical regions and early infections may lead to large scale epidemics in a short period (Bhavani et al., 2019). Severe stem rust pressure can affect tillering (Rowell and Roelfs, 1976), plant growth by reducing the photosynthetic area, nutrients and water supply, and their transportation in the plant system (Roelfs, 1985). The two most severe epidemics of Europe occurred in Scandinavia (1951) and central Europe (1932) causing nationwide yield losses up to 20–33% (Zadoks and Bouwman, 1985). During, 1948–56 a few severe epidemics were evident in China due to higher than average temperatures and frequent rains which created a conducive environment for the infections. Other historical events of wheat losses due to this disease occurred in Australia during 1973 (AUD 100–200 million loss, McIntosh et al., 1995), United States (North Dakota) during 1919–1923 (1.95 million tons loss, Roelfs, 1978) and 1950–1955 (3.74 million tons, Roelfs, 1978), New Zealand in 1980 (Beresford, 1982), continental Europe, and the Indian subcontinent (Chaves et al., 2013; Singh et al., 2015). The last major stem rust epidemics occurred in Ethiopia in 1993 and 1994 (Shank, 1994). In a major outbreak in 1998, a new race (Ug99) of stem rust pathogen virulent to *Sr31* was first reported in Uganda (Pretorius et al., 2000), and its lineages (TKTTF) have caused 100% yield losses on susceptible wheat cultivars during 2013–14 in Ethiopia (Singh et al., 2015). It is still considered to be a potential threat for Kenya, Ethiopia, Yemen, the Middle East, and South Asia. The overall global crop losses associated with Pgt alone were estimated to be approximately USD 53.7 billion per annum (Pardey et al., 2013).

## Identification and Mapping of Rust Resistance Genes

Breeding for genetic resistance assists in developing rust-resistant wheat varieties. Genetic resistance can be categorized in three groups: 1. monogenic resistance (resistance governed by a single gene), 2. oligogenic resistance (resistance governed by few genes) and 3. polygenic resistance (resistance governed by many genes with additive effects) (Russel, 1978). Based on the resistance exhibited, individual rust resistance genes have been named as major genes and minor genes (Russel, 1978). In pathological terms, rust resistance could be distinguished as horizontal resistance and vertical resistance. Horizontal resistance is race non-specific resistance and effective against genetic variants of a rust pathogen. Vertical resistance is effective against certain pathogen races, so it is also called race-specific resistance (Van der Plank, 1963). The term durable resistance is frequently used instead of non-race specific or horizontal resistance to describe long-lasting resistance. The durability does not mean that resistance is active against all genetic variants of a pathogen but simply that the resistance was effective for a long time (Johnson and Law, 1975). Durable resistance may be conferred by a single gene or by combinations of many genes (McIntosh and Watson, 1982). The theory of gene-for-gene relationship implies that for each resistance gene in the host, there is a corresponding and specific avirulence gene in the pathogen (Flor, 1956). This relationship shows two reactions: 1. Incompatible reaction and 2. Compatible reaction. The incompatible reaction can be evident when a pathogen carries avirulence alleles corresponding to the host’s resistant alleles which results in a resistance response. Compatible reaction reveals a susceptible response when a pathogen carries virulence alleles to a susceptible host. The pathogen could carry many avirulence genes; each one corresponding to a resistance gene of a cultivar (Person, 1959). The gene-for-gene hypothesis defines existing diversity in physiological races of a pathogen and resistant genes among the relevant host species.

Wild progenitor and related species of wheat constitute an immense reservoir of genetic variation important for several economic traits including resistance to biotic stresses. This had resulted in an opportunity to breeders for transferring resistance genes from wild species to modern wheat cultivars through inter-specific hybridization (Bansal et al., 2017). Over two-hundred twenty rust resistance genes comprising 60 stem rust resistance, 79 leaf rust resistance genes and 83 stripe rust resistance genes have been formally named and catalogued using bi-parental populations in wheat (McIntosh et al., 2017; Li et al., 2020a). During the last decade, discovery and precise mapping of rust resistance genes expedited, and robust breeder-friendly linked-markers have been reported for several genes (**Tables 1A**, **B**). This was largely possible due to the availability of high throughput genotyping platforms including DArTSeq (www.diversityarrays.com), i-select 90K SNP Infinium array (Wang et al., 2014) and genotyping-by-sequencing approaches (Poland and Rife, 2012).

**Table 1A:** Seedling rust-resistant genes and their linked markers.

Seedling rust-resistant genes	Location	Donor	Linked Marker(s)	References
*Yr15*	1BS	A WEW accession G25	*uhw264* and *uhw259*	Klymiuk et al., 2018
*Yr51*	4AL	A wheat landrace AUS27858	*owm45F3R3* and *sun104*	Randhawa et al., 2014
*Yr53*	2BL	A durum wheat accession PI 480148	*wmc441* and *LRRrev/NLRRrev350*	Xu et al., 2013
*Yr60*	4AL	A wheat line Almop	*wmc219* and *wmc313*	Herrera-Foessel et al., 2014a
*Yr61*	7AS	A Chinese cultivar Pindong34	*STS5467* and *STS5765b*	Zhou et al., 2014a
*Yr63*	7BS	A wheat landrace AUS 27955	*IWB33120* and *IWB52844*	McIntosh et al., 2017
*Yr70/Lr76*	5DS	An introgressed line IL393-4 carrying gene from *Ae. umbellulata*	*gwm190*	Bansal et al., 2017
*Yr73*	3DL	An AUS cv. Avocet R (*YrA* resistance was inherited by two complementary dominant genes *Yr73* and *Yr74*)	*DArT markers 116719[F]0*	Dracatos et al., 2016
*Yr74*	5BL	An AUS cv. Avocet R (*YrA* resistance was inherited by two complementary dominant genes *Yr73* and *Yr74*)	*DArT markers 1091695[F]0*	Dracatos et al., 2016
*Yr81*	6AS	Wheat landrace AUS27430	*KASP_3077* and *gwm459*	Gessese et al., 2019
*Yr82*	3BL	Wheat landrace AUS27969	*sun KASP_300* and *sunKASP_8775*	Pakeerathan et al., 2019
*Lr16/Sr23*	2BS	A Canadian spring wheat line BW278 (AC Domain*2/Sumai3)	*2BS_5175914_kwm847*	Kassa et al., 2017
*Lr23*	2BS	A common wheat genotype BT-Schomburgk selection and durum wheat cultivar Tamaroi	*sunKASP_16* and *sunKASP_47*	Chhetri et al., 2017
*Lr24/Sr24*	3DL	A wheat variety Agent carrying a translocation from *Agropyron elongatum*.	*Sr24#12-F* and *Sr24#12-R*	Mago et al., 2005
*Lr37/Sr38/Yr17*	2AS#2NS	*T. ventricosum* accession #10	*VENTRIUP* and *LN2*	Helguera et al., 2003
*Lr52/Yr47*	5BS	A wheat landrace Aus28183	*sun180*	Qureshi et al., 2016
*Lr65*	2AS	A swiss spelt wheat ‘Altgold Rotkorn’	*gwm614* and *barc124*	Mohler et al., 2012
*Lr71*	1B	A swiss spelt wheat cv. Altgold Rotkorn	*gwm18* and *barc187*	Singh et al., 2001
*Lr72*	7BS	A Mexican durum wheat cultivar Atil C2000	*wmc606*	Herrera-Foessel et al., 2013
*Lr73*	2BS	A wheat genotype Morocco	*wPt-4453* and *wPt-8235*	Park et al., 2014
*Lr79*	3BL	A durum wheat landrace Aus26582	*sun786*	Qureshi et al., 2018c
*Sr13*	6AL	An emmer wheat Khapli and Ethiopian accession ST464	*EX24785*	Zhang et al., 2017
*Sr26*	6AL	A wheat genotype Avocet ‘S’ carrying an alien segment 6Ae#1L from *Ag. elongatum*	*sunKASP_224* and *sunKASP_225*	Qureshi et al., 2018b
*Sr52*	T6AS·6V#3L	A wheat-*Dasypyrum villosum* disomic addition line DA6V#3	*BF201083* and *BE490365*	Qi et al., 2011
*Sr53*	5DL	TA5599 (T5DL-5MgL·5MgS) carrying an alien segment from *Aegilops geniculata*	*BE443021/MboI* and *BE442814/HaeIII*,	Liu et al., 2011
*Sr54*	2D	A winter wheat cultivar Norin40	*barc228* and *wmc181*	Ghazvini et al., 2012
*Sr60*	5A	*T.monococcum* accession PI 306540	*ucw530* and *ucw540*	Chen S. et al., 2020

**Table 1B:** Adult plant rust-resistant genes in wheat and their linked markers.

Adult plant rust resistance genes	Location	Donor	Linked Marker(s)	References
*Yr48*	5AL	Synthetic wheat PI610750	*wmc727* and *cfa2149*	Lowe et al., 2011
*Yr52*	7BL	A wheat germplasm PI 183527 carrying HTAP resistance	*barc182* and *wgp5258*	Ren et al., 2012
*Yr54*	2DL	A wheat line Quaiu3	*wpt-667162, wpt-667054* and *gwm301*	Basnet et al., 2014
*Yr56*	2AS	A landrace AUS 91575 and Wollaroi (AUS 99174)	*sun167* and *sun168*	McIntosh et al., 2017
*Yr59*	7BL	Iraqi germplasm PI 178759	*wgp5175* and *barc32*	Zhou et al., 2014b
*Yr62*	4BL	Spring wheat germplasm PI 192252	*gwm192* and *gwm251*	Lu et al., 2014
*Yr68*	4BL	Undesignated International Nursery ex New Zealand 03.25 (AGG91587WHEA)	*IWB74301* and *IWA4640*	McIntosh et al., 2017
*Yr71*	3DL	An Australian wheat variety Sunco	*gwm114b, KASP_16434, KASP_17207* and *KASP_20836*	Bariana et al., 2016
*Yr75*	7AL	An Australian wheat variety Axe	*IWB34640* and *cfa2040*	McIntosh et al., 2017
*Yr77*	6DS	A common wheat PI322118	*IWA167* and *barc54*	McIntosh et al., 2017
*Yr78*	6BS	A common wheat PI519805	*IWA7257* and *IWA4408*	Dong et al., 2017
*Yr79*	7BL	A common wheat PI182103 carrying HTAP resistance	*barc72* and *wmc335*	Feng et al., 2018
*Yr80*	3BL	A wheat landrace AUS27284	*sunKASP_65624* and *sunKASP_53113*	Nsabiyera et al., 2018
*Lr68*	7BL	A common wheat cultivar Parula	*cs7BLNLRR-F/cs7BLNLRR-R*	Herrera-Foessel et al., 2012
*Lr74*	3BL	A common wheat genotype BT-Schomburgk selection	*GBS2256311* and *IWB69699*	McIntosh et al., 2017
*Lr75*	1BL	A Swiss winter wheat cultivar ‘Forno’	*B2g38480* and *B2g38640*	Singla et al., 2017
*Sr56*	5BL	A Swiss winter wheat Arina	*sun209* and *sun320*	Bansal et al., 2014
*Sr55/Lr67/Yr46*	4DL	A PAK landrace PI 250413	*TM 4* and *TM10*	Moore et al., 2015
*Sr57/Lr34/Yr18*	7DS	A wheat genotype Parula (PI58548)	*csLV34*	Lagudah et al., 2009
*Sr58/Lr46/Yr29*	1BL	A wheat cultivar Pavon76	*SNP1_Lr46G22*	Lagudah, personnel communication
*Sr2/Lr27/Yr30*	3BS	A wheat cultivar Hope	*csSr2*	Mago et al., 2011

### Pleiotropic Adult Plant Resistances

Four APRs *Lr27*/*Yr30*/*Sr2, Lr34/Yr18/Sr57, Lr46/Yr29/Sr58*, and *Lr67/Yr46/Sr55* harboring multiple pathogen resistance have been identified (McIntosh et al., 1995; **Table 1B**). These genes resist devastation incurred by three rusts and powdery mildew caused by *Blumeria graminis f.* sp. *tritici*. The response of *Lr34, Lr46*, and *Lr67* is well characterized by their partial resistance to each of these pathogens and a phenotypic marker, leaf tip necrosis (Ltn) (Lillemo et al., 2008; Herrera-Foessel et al., 2014b). The pleiotropic APR *Lr34* has a comparatively higher level of rust resistance than other APRs (Lillemo et al., 2008; Yang et al., 2013). The APRs *Lr34/Yr18/Sr57/Pm38/Ltn1* and *Lr46/Yr29/Sr58/Pm39/Ltn2* have expressed additive gene interaction in combination and is frequently used in CIMMYT breeding programs (Lillemo et al., 2008; Lillemo et al., 2013). Unlike ASRs, the class of APR does not rely on a gene-for-gene hypothesis and partial resistance against a range of pathotypes (Dodds et al., 2010). Cloning of *Lr34/Yr18/Sr57/Pm38/Ltn1* and *Lr67/Yr46/Sr55/Pm46/Ltn3* (Krattinger et al., 2009; Krattinger et al., 2011; Moore et al., 2015) has demonstrated their role in conferring disease resistance in cereals like Sorghum (Schnippenkoetter et al., 2017), Durum wheat (Rinaldo et al., 2017), Rice (Krattinger et al., 2016) and Barley (Risk et al., 2013; Milne et al., 2019). It is also indicated that the resistance response of *Lr34res* and *Lr67res* occurs due to gain of resistance function through alteration in two amino acids in each of ATP-Binding Cassette (ABC) transporter and Hexose (sugar) Transporter Protein (STP), respectively (Krattinger et al., 2011; Moore et al., 2015). The molecular mechanism of few of these genes is not well understood as compared to NLR genes, and the mode of action of Lr34 transporter and molecules it transports is completely unknown (Rinaldo et al., 2017). There are three possible scenarios which may explain the mode of action of Lr67res; First, is altered carbon partitioning caused by Lr67res to limit nutrients to the pathogen, secondly, altered hexose/sucrose ratio triggering defense response and thirdly reduced pathogen growth due to unknown mechanism triggered by altered function of Lr67res (Milne et al., 2019).

### Bi-Parental Mapping *vs* Genome-Wide Association Studies (GWAS)

Gene postulation is a classical approach to identify new genes phenotypically, using rust infection type response, multi pathotype test for gene postulation and differential sets. There are two alternative approaches to identify new rust resistance genes. The first approach includes the identification of donor parents expressing a high level of rust resistance and developing bi-parental mapping populations by crossing donor lines with susceptible lines. Genetic studies at F_2_ and F_3_ generations help us to estimate the number of genetic factors controlling the trait and their interactions in controlling resistance. Selective genotyping at the early segregating generations and/or advanced stages like F_5_ and F_6_ generations assists in identifying genomic region(s) underpinning rust resistance in the population (Bansal and Bariana, 2017). Bulk segregant analysis (BSA) is the most commonly used, efficient, and rapid method to identify markers closely linked to a specific gene or any genomic regions (Michelmore et al., 1991) by genotyping a pool of DNA from selected individuals with two extreme phenotypes and the contrasting parents. Then the allelic frequency differences between the bulks will be analyzed and compared with the phenotype to ascertain the possible linkage between the markers and trait of interest (Kthiri et al., 2018). Earlier, in the decade, SSR markers evenly spanning the wheat chromosomes were very frequently used to screen for polymorphic markers but, it was time-consuming and laborious as it was done manually. In recent times, the integration of next-generation sequencing-based 90K SNP arrays with BSA approach has quickened the identification of polymorphic markers and chromosomal location of major genes or the QTLs (Abe et al., 2012; Ramirez-Gonzalez et al., 2015; Qureshi et al., 2016; Kthiri et al., 2018; Wu et al., 2018; Pakeerathan et al., 2019). The BSA approach has also been combined with RNA-Seq to identify gene associated SNPs, NLR exome capture and sequencing (also called as MapRenSeq) to define NLRs associated with resistant genes (Narang et al., 2020) and NGS-based SLAF-seq to locate the gene of interest/QTL (Yin et al., 2018). However, the mapping of disease resistance genes and quantitative trait loci (QTL) through traditional bi-parental mapping approaches is restricted only to the limited diversity present in the parents and the less number of recombination events which results in low map resolution.

Alternatively, the second approach, Genome-wide association studies (GWAS) is primarily focused on quickly screening germplasm collection for genes of interest and to capture historical recombinations to achieve high-resolution mapping at a specific locus. As an advantage over mapping in bi-parental populations, this approach makes use of already existing natural populations, accounts for greater allelic diversity among the diverse individuals at a given locus and uses the recombination events that occur throughout the evolutionary process of germplasm (Yao et al., 2019). To excel in GWAS analysis, as a prerequisite, this approach would need a diversity panel constituting accessions and/or elite lines with similar photoperiod requirements and adaptation (Yu and Buckler, 2006). It has played a remarkable role in dissecting various complex traits in cereals (Yu et al., 2011; Juliana et al., 2015), and several of major and minor quantitative trait loci (QTL) relying on significant marker-trait associations (MTAs) for rust resistance have been identified through this approach (Rosewarne et al., 2012; Li et al., 2014; Zhang et al., 2014; Maccaferri et al., 2015; Juliana et al., 2018) but, none of the named rust genes has been identified through this approach until now. Moreover, several of these novel QTLs have not been validated and functionally characterized which have limited their use in breeding programs (Juliana et al., 2018). Though advances in statistical analysis and genotyping platforms have facilitated in ruling out the false-positive MTAs and rapid discovery of candidate genes using genome references (Bradbury et al., 2007; Zhang et al., 2010; Tang et al., 2016), population structure is still a critical concern and can be overcome by using mixed models to integrate genetic relatedness.

## Application of Genomics Tools in Mining and Isolation of Rust Resistance Genes

### DNA Marker Technology in Wheat

DNA markers reveal allelic variations among individual organisms or species. In wheat research, DNA marker technology has been used in tracing targeted genes. This technique is consistent, mostly neutral/free from environmental variations and does not affect the developmental stage of the plant as it is based on variations in DNA sequences (Collard et al., 2005). Some morphological and biochemical markers are also being used to identify lines carrying durable genes, for instance, pseudo-black chaff and high-temperature-induced seedling chlorosis linked with *Sr2* (Slinkard and McIntosh, 1998), red-glume gene (Rg1) linked with *Yr10* and leaf-tip necrosis (*Ltn*) linked with *Lr34, Lr46, Lr67*, and *Lr68* (Lagudah et al., 2006; Herrera-Foessel et al., 2012; Herrera-Foessel et al., 2014b). The DNA marker technology offers a major advantage in plant breeding by exploring modern genomics tools. The Polymerase chain reaction (PCR)-based markers have been designed and applied to detect marker-trait association across seven groups of wheat chromosomes. The DNA markers namely Restriction fragment length polymorphism (RFLP), Sequence Characterized Amplified Region (SCAR), Sequence Tagged Site (STS), Simple Sequence Repeat (SSR), and Cleaved Amplified Polymorphic Sequences (CAPS) were widely explored as valuable tools for wheat improvement. Gene mapping in wheat was initiated using conventional genetic markers RFLPs (Anderson et al., 1992) and SSRs (Röder et al., 1998). Later, additional PCR-based markers like RGAP, CAPS, EST, STS, AFLP, and RAPD have been implemented in the mapping of disease resistance genes (Bariana et al., 2001). The consensus map of 2004 SSRs has been used to integrate conventional markers to some extent for drafting an integrated map of the identified gene/loci using cMap (Somers et al., 2004; Rosewarne et al., 2013). Sanger sequencing accelerated the identification of the single base pair variations (Wang et al., 1998) and helped to replace gel-based marker systems with robust and bi-allelic single nucleotide polymorphisms (SNPs). This SNP-based DNA marker has enhanced its implementation to detect allelic variation in targeted DNA fragments. The advantage of MAS (Marker Assisted Selection) is not only rapid detection assays for the presence of the gene but also enables gene pyramiding strategies wherein it is difficult to detect gene combinations due to epistatic/masking effect if race-specific genes.

### Next-Generation Sequencing (NGS) Platforms

The NGS platforms have revolutionized genetics and genomics studies in crop plants. Several NGS-based approaches played a significant role in allele discovery and genotype-by-sequencing (GBS) through thousands of markers simultaneously to precisely cover the entire crop genome (Davey et al., 2011; Liu H. et al., 2014). In wheat, NGS technology enables detecting the abundance of DNA markers within a short period. The size of the wheat genome is huge (~16GB—around five times bigger than the human genome), more complex, and contains a high percentage of repetitive elements. Several marker development methods have been reported, including RRLs (reduced-representation libraries), CRoPS (complexity reduction of polymorphic sequences), RAD-seq (restriction-site associated DNA sequencing), SBP (sequence-based polymorphic marker technology), MSG (low coverage multiplexed shotgun genotyping), and GBS (genotyping-by-sequencing) to reduce the complexity (Yang et al., 2012). Additionally, GBS pipeline was also developed to reduce the complexity of the wheat genome. The RAD-seq and GBS were explored to target cutting sites after restriction digestion to avoid further redundancy in the genome (Poland et al., 2012). High throughput genotyping platforms including DArT-Seq, SNPs, GBS markers, and population-specific tGBS (targeted genotyping-by-sequencing) have expedited the precise mapping of genomic regions underpinning rust resistance (Qureshi et al., 2018b; Nsabiyera et al., 2020).

### Discovery of SNPs Using a Genotyping Array

Discovery of single nucleotide polymorphisms (SNPs) in wheat has been expedited through the advances in genomics resources. Cavanagh et al. (2013) developed a high-throughput SNP genotyping array called i-select 9K SNP Infinium Bead chip Assay using 2,994 worldwide wheat accessions. Later, a high-density SNP map was developed after incorporating the first map and customized as i-select 90K SNP Infinium array covering seven groups of wheat chromosomes (Wang et al., 2014). These linked SNPs would facilitate to overcome, a major bottleneck associated with the application of DNA markers to characterize a wide range of germplasm (Yang et al., 2012). This high-density SNP map comprises nearly 90,000 gene-linked SNPs to characterize genetic variability in tetraploid and hexaploid wheat (Wang et al., 2014). Similarly, the GBS approach was utilized to characterize over 40,000 CIMMYT wheat germplasms as part of Seeds of Discovery (SeeD) initiative (Li et al., 2015). A consensus map of GBS markers was constructed to detect potentially new APR linked with stripe rust, leaf rust, and stem rust resistance and validate known genes/QTL through aligning them on wheat genome reference (IWGSC Refseq v 1.0; Appels et al., 2018). Identified trait-linked SNPs can be converted into Kompetitive allele-specific PCR assay (KASP) using PolyMarker, an automated bioinformatics pipeline (http://www.polymarker.info). The same SNPs are available in different wheat chromosomes at various positions due to its evolutionary nature and diploidization phenomenon. PolyMarker can design SNP-based KASP of the known genome-specificity using IWGSC RefSeq v1.0 (Ramirez-Gonzalez et al., 2015). Developing KASP markers cannot be guaranteed their utility in marker-assisted selection. Mendelian inheritance of individual KASP in a bi-parental mapping population and linkage with phenotypic data can ensure their application in the wheat breeding program.

### Challenges to Draft Consensus Maps of Rust Resistance Genes

Several new resistance loci and robust markers of reported genes were developed and explored in gene pyramiding (Bariana et al., 2007; Wessels and Botes, 2014; Randhawa et al., 2019). High level of redundancy in these identified loci and common MTAs was evident among different mapping studies. A consensus map using public database was drafted for stripe rust resistance on wheat chromosomes with the help of flanking markers to highlight around 140 stripe rust resistance loci and project redundant loci identified in over 30 bi-parental studies (Rosewarne et al., 2013). This study reduced the number of genomic regions conferring stripe rust resistance and laid a platform for further works. Later, Maccaferri et al. (2015) developed an integrated map of stripe rust resistance regions after incorporating significant QTLs identified in ten experiment-wise GWAS studies, 56 *YR* genes and 169 mapped QTLs. An “iterative maps compilation” tool in Biomercator v4.2 was explored to integrate the consensus map of SSRs (Somers et al., 2004), Synthetic × Opata ITMI BARC SSR map (Song et al., 2005), i-select 90K SNP Infinium array, Synthetic × Opata DH GBS map (Saintenac et al., 2013a) and Diversity Array Technology (DArT) map (http://www.diversityarrays.com) to create an integrated map (Maccaferri et al., 2015). This consensus map facilitated in the integration of different marker systems like RFLP, DArT, SSR along with flanking robust SNPs and align them together using published genetic map. This information is available on the GrainGenes webpage (https://wheat.pw.usda.gov/GG3/). Similar approaches have been adapted to develop stem rust resistance loci consensus map of detected in 24 bi-parental mapping populations, three association panels, two backcross populations (Yu et al., 2014), and QTL conferring leaf rust and powdery mildew resistance in wheat (Li et al., 2014).

Several genomic loci are well known to harbor more than one gene, but these integrated maps did not allow further delineation of these regions. For instance, wheat chromosome 5A carries two genes *Yr34* and *Yr48* which were later confirmed as allelic or the same (Qureshi et al., 2018a). Gao et al. (2015) attempted to enrich region of SrCad (source- Canadian wheat cultivar AC Cadillac) and *Sr42* (Norin 40) using GBS and 90K SNP array approach which placed both these genes at the same locus on chromosome 6DS. Moreover, both genes shared a common donor, BW553 and resistance to TTKSK isolate which also suggested that both genes are the same or allelic. Differential expression of AC Cadillac to non-TTKSK Pgt races compared to Norin 40 is due to the existence of background genes like *Lr34*. Cloning of *Rph15* in barley confirmed that *Rph15* and *Rph16* are allelic or the same (Chen C. et al., 2020).

Integrating wheat genome reference (IWGSC RefSeq v 1.0) with the previously published genetic map of rust resistance loci was a major challenge; however, the new genetic map viewing software, Pretzel, was designed to fix the issues. This software is currently being explored to align trait-linked SNPs, DArTseq, and GBS markers with multiple SNP platforms of available wheat and related genomic resources like *Triticum dicoccoides*, *T. turgidum*, *Aegilops tauschii*, *T. urartu*, *Brachypodium_distachyon*, *Hordeum vulgare*, and *Oryza sativa* (Keeble-Gagnere et al., 2019; http://plantinformatics.io/mapview). It assists in the positioning of various genomic regions of the different populations using IWGSC RefSeq v1.0. It would be a milestone achievement in differentiating rust resistance genes/MTAs underlying in similar genomic regions.

### Rapid Rust Resistance Gene Cloning Methods

In plants, most of the disease resistance genes are race-specific and contain the Nucleotide-binding site (NBS) and LRR (Leucine-rich repeat) domains. These genes are believed to be regulated by NBS domains through signal transduction, and specific sites of corresponding pathogen virulence genes are recognized by LRR domains (Gill et al., 2015). Six stripe rust resistance genes, *Yr5/YrSP, Yr7, Yr10, Yr15, Yr36*, and *YrAS2388R* (Fu et al., 2009; Liu H. et al., 2014; Klymiuk et al., 2018; Marchal et al., 2018; Zhang et al., 2019), four-leaf rust resistance genes *Lr1, Lr10*, *Lr21*, and *Lr22a* (Feuillet et al., 2003; Huang et al., 2003; Cloutier et al., 2007; Thind et al., 2017), and nine major stem rust resistance genes, *Sr33, Sr35, Sr50, Sr22, Sr45, Sr13, Sr46, Sr21*, and *Sr60* (Periyannan et al., 2013; Saintenac et al., 2013b; Mago et al., 2015; Steuernagel et al., 2016; Zhang et al., 2017; Arora et al., 2018; Chen et al., 2018; Chen S. et al., 2020), and two pleiotropic APR (*Lr34/Yr18/Sr57* and *Lr67/Yr46/Sr55*) (Krattinger et al., 2009; Moore et al., 2015) have been cloned (**Table 2**).

**Table 2:** List of cloned rust resistance genes in wheat.

**Gene/Locus**	**Chromosome**	**Approach**	**Major/Minor gene**	**Protein encoded**	**References**
*Yr5/YrSP*	2B	MutRenSeq	Major	NBS-LRR	Marchal et al., 2018
*Yr7*	2B	MutRenSeq	Major	NBS-LRR	Marchal et al., 2018
*Yr10*	1BS	Map-based cloning	Major	NBS-LRR	Liu W. et al., 2014
*Yr15*	1BS	Map-based cloning	Major	A wheat tandem kinase 1	Klymiuk et al., 2018
*YrAS2388R*	*4DS*	Map-based cloning	Major	CC-NBS-LRR	Zhang et al., 2019
*Lr1*	5DL	Map-based cloning	Major	CC-NBS-LRR	Cloutier et al., 2007
*Lr10*	1AS	Map-based cloning	Major	CC-NBS-LRR	Feuillet et al., 2003
*Lr21*	1DS	Map-based cloning	Major	CC-NBS-LRR	Huang et al., 2003
*Sr13*	6AL	map-based cloning	Major	CC-NBS-LRR	Zhang et al. (2017)
*Sr21*	2A^m^L	map-based cloning	Major	CC-NBS-LRR	Chen et al. (2018)
*Sr22*	7A	MutRenSeq	Major	CC-NBS-LRR	Steuernagel et al., 2016
*Sr33*	1D	Map-based cloning	Major	CC-NBS-LRR	Periyannan et al., 2013
*Sr35*	3AL	Map-based cloning	Major	CC-NBS-LRR	Saintenac et al. (2013b)
*Sr45*	1D	MutRenSeq	Major	CC-NBS-LRR	Steuernagel et al., 2016
*Sr 46*	2DS	AgRenSeq	Major	coiled-coil NLR protein	Arora et al., 2018
*Sr50*	1RS	physical mapping, mutation and complementation	Major	CC-NBS-LRR	Mago et al. (2015)
*Sr60*	5AmS of T. monococcum	Map-based cloning	Major	A wheat tandem kinase 2	Chen S. et al., 2020
*Yr36*	*6BS*	Map-based cloning	Minor	Kinase-START gene	Fu et al., 2009
*Lr22a*	2DS	TACCA	Minor	CC-NBS-LRR	Thind et al., 2017
*Lr34/Yr18/Sr57*	7DS	Map-based cloning	Minor	ABC transporter	Krattinger et al., 2009
*Lr67/Yr46/Sr55*	4DL	Map-based cloning	Minor	Hexose transporter	Moore et al., 2015

Map-based cloning includes fine mapping of an individual gene, discovery of candidate genes followed by validation of these genes using mutants and available genomics resources (Jander et al., 2002; Thind et al., 2017; Zhang et al., 2019). The first study towards the map-based cloning of Rpg1 and Rpg4 using rice as an inter-genomic mapping vehicle was reported in Barley (Kilian et al., 1997). Many genes of economic importance in cereal crops, most notably, *chloronerva*, a gene involved in the uptake of iron in higher plants encoding *Nicotianamine synthase* (Ling et al., 1999), beta and old-gold (og) color mutations in tomato (Ronen et al., 2000), *Rf-1* in rice (Komori et al., 2004), Soybean *Maturity Locus E3* (Watanabe et al., 2009), *TTG1* homolog in Brassica (Zhang et al., 2009), and *AvrLm6* avirulence gene in *Leptosphaeria maculans* (Fudal et al., 2007), *Lr10* (Feuillet et al., 2003), *Lr21* (Huang et al., 2003), and *Lr1* (Ling et al., 2003) were isolated using this strategy. The main problem associated with map-based cloning is lack of sufficient DNA markers to create a fine map in different crops (Drenkard et al., 2002). Besides, it was essential to construct a physical map, identify markers, and reach close to the gene through chromosome walking. It was then followed by isolation, transformation, complementation, and determination of the sequence of the entire region of interest in the absence of any reference sequence of the wild-type DNA (Jander et al., 2002).

In crops with large genomes like wheat, complexity reduction is very important to clone target genes more quickly and efficiently. A recently developed molecular tool, MutChromSeq (Mutgenesis Chromosome flow sorting and short-read Sequencing), works based on mutagenesis followed by flow sorting of chromosomes and their sequencing to identify the possible induced mutations. This approach does not require a similar gene in bait library and hence enables reference-free forward genetics to open up the pan-genome to functional genomics (Sanchez-Martin et al., 2016; Zhang et al., 2020). This rapid cloning approach which would need 18–24 months for mutagenesis and screening, approximately one month for chromosome flow sorting, sequencing and bioinformatics were successfully described for cloning of barley *Eceriferum*-*q* gene and wheat *Pm2* gene (Sanchez-Martin et al., 2016). The first leaf rust resistance gene *Rph1* (*Rph1*.*a*) on chromosome 2H from cultivated barley (*Hordeum vulgare*) cv Sudan was also rapidly cloned by combining this approach with genetic mapping. The successful application of this approach would need prior information on, chromosome location of the target gene (Dracatos et al., 2019; Zhang et al., 2020), large mutant populations to identify the target genes among five to six mutants (Mago et al., 2017; Dinh et al., 2020) and isolation of individual chromosomes (Periyannan, 2018).

As an alternate approach to MutChromSeq, TACCA makes use of flow sorting of chromosomes, next-generation sequencing, and cultivar specific *de-novo* chromosome assembly. Using this approach a broad-spectrum leaf-rust resistance locus *Lr22a* (encodes an intracellular immune receptor homologous to the Arabidopsis thaliana RPM1 protein) was cloned with molecular marker information and ethyl methanesulfonate (EMS) mutants (Thind et al., 2017).

There are other cloning strategies like MutMap (mutational mapping) which involve mutagenesis, sequencing, and mapping to identify SNPs between wild-type and homozygous mutants and then to narrow down to the region having gene of interest (Li et al., 2020b). Until recently, this approach was considered to be applicable only in crops with small genomes like rice (Abe et al., 2012), but it was demonstrated to successfully map and clone *Ms1* from allohexaploid bread wheat using F_2_ plants derived from the progeny of heterozygous *ms1e* mutants (Wang et al., 2017). Still, this is a better choice when the genome is small, and high-quality reference genome is available.

A three-step pipeline including chemical mutagenesis (Mut), exome capture and sequencing (RenSeq) techniques was developed for the rapid isolation of genes (Steuernagel et al., 2016). Here, ‘Mut’ confers mutagenesis that is a technique to produce random mutations or nucleotide substitutions in the DNA sequence by treating the parent material with mutagens like EMS (Ethyl Methane Sulfonate CH_3_SO_3_C_2_H_5_) or Sodium Azide (NaN_3_). This substitution results in GC to AT transition and can cause loss of function of the resistance gene. An initial kill-curve analysis is used to decide the optimum dose of mutagen to render 50% mortality and reduced growth (Periyannan et al., 2013). The ‘RenSeq’ technique relies on the sequence information of known resistance genes and uses as bait to capture similar gene fragments in closely related crops and species (Jupe et al., 2013; Steuernagel et al., 2016). In plants, most of the known disease resistance genes belong to NLRs, kinases (receptor-like kinase, receptor-like proteins, wall-associated kinase *etc*) and/or transporters families (Klymiuk et al., 2018). Two stem rust resistance genes (*Sr22* and *Sr45)* were cloned using this technique. In MutRenSeq, NLR gene families are targeted, as it is the major class of R gene family conferring disease resistance in plants. The procedure includes: (1) Bait library construction containing RNA probes with more sequence identity with predicted NLR genes, (2) Preparation of template DNA from the accessions or germplasm conferring disease resistance, (3) Gene capture/hybridization using bait library in PCR based hybridization reaction, (4) Next-generation sequencing of captured and enriched fragments to predict NLR gene structures, (5) Assembly of NBS-LRR reads and their comparison and (6) Validation of candidate genes. MutRenSeq is a rapid and accessible to several organisms including wheat (Steuernagel et al., 2016). The major limitations of this technique are its restriction to discover only the NLR gene, a reference pan-genome and vital changes due to mutagenesis (Dinh et al., 2020).

AgRenSeq (Association genetics with R-gene enrichment sequencing) or speed cloning has been developed to align GWAS platform (to utilize diverse genome-wide natural variation) and the RenSeq. Unlike map-based cloning and MutRenSeq, this approach does not rely on bi-parental mapping populations or mutagenesis. For instance, AgRenSeq approach was successfully demonstrated to clone R gene, *Sr46* and to identify the candidate gene sequence for *SrTA1662* using a diverse panel of *Ae. tauschii ssp.strangulata* (Arora et al., 2018). It explores a pool of diverse wild relatives carrying many resistance genes as a result enabling the cloning of multiple genes at the same time (Dinh et al., 2020).

## Breeding Strategies to Develop Rust-Resistant Wheat Varieties

Relentless efforts to enhance modern wheat cultivars for rust resistance is of primary importance to many breeding programs which come with a challenge of, not to compromise with yield and quality traits. A rust-resistant hard wheat variety, “Marquillo” developed in 1918 (Hayes et al., 1920) had considerable resistance to rust, but it lacked the flour quality, so it could not become popular in North Dakota (Stoa, 1945). Similarly, a highly rust-resistant line named, “Hope” (McFadden, 1930) which had poor yield potential under drought and heat stress conditions. However, the popular rust-resistant wheat cultivar, “Thatcher” released in 1934 (Hayes et al., 1936) carried multiple stem rust resistance genes and provided useful resistance against stem rust in North America (Singh et al., 2004b) occupying 60% of the area during that period under hard red spring wheat in North Dakota. Being a most preferred strategy of rust control, systematic breeding for genetic resistance in wheat started when the inheritance of yellow rust resistance was demonstrated following Mendelian principles of heredity (Biffen, 1905). The resistance controlled by a single locus was considered to be sufficient for complete control of the rust pathogens, until the discoveries of Flor (1956), when the incompatibility of host and pathogens was entirely said to be based on corresponding gene present in the organism and the resistance governed by a single gene need not be always durable. In the 20^th^ century, though, breeders were aware of possible single mutations in pathogens’ Avr genes, could result in new virulent pathotypes, still most of the breeding programs adopted single gene pyramiding into cultivars as major “breeding strategy”. This could be due to ease in the screening of breeding materials at the seedling stage and high heritability and clear phenotype of the resistance expressed by major genes (Pink and Hand, 2002).

There are multiple approaches, conventional and/or molecular, singly or in combination, being considered to develop wheat varieties with durable rust resistance around the globe. Traditionally, using conventional approaches, the available germplasm and breeding materials are screened at seedling and adult plant stage, to phenotypically identify resistant wheat lines and then to involve them in hybridization programs as one of the parents. Till today, there are several breeding teams with no access to advanced MAS tools have successfully adopted this approach to breeding future rust-resistant wheat varieties. This makes the best use of shuttle breeding, wherein, the breeding lines are grown in one or more off-season locations with congenial environments for all the three individual rust diseases. Due to the cultivation of host crops throughout the year, these offseason environments become the evolution hubs for the new races, which helps them to prolifer and multiply continuously. Besides, these environments will also assist breeder to carefully select wheat lines which show consistent resistance across the years and locations with a large number of naturally occurring pathotype mixtures in the environment.

Breeding strategies to improve wheat for seedling and adult plant resistance may vary usually due to their mode of inheritance which is either qualitative and/or quantitative. Among the many breeding methodologies available to improve a particular trait in crop plants, pureline selection, modified pedigree method, bulk breeding, recurrent selection, backcross breeding, mutation and single seed descent methods of breeding are applied to breed wheat for rusts resistance. Pureline selection improved the agronomical feature of several landraces resulting into popular wheat varieties namely Sharbati, White Pissi, Chandausi and Lal Kanak in India. The wheat variety Marquis was developed as a selection from hybridization between an early ripening Indian wheat “Hard Red Calcutta” and “Common Fife” in 1909. Due to early ripening, it escaped the injury caused by heat and rust to become a prominent variety for several years in Western North Dakota (Stoa, 1945). Breeding through a modified pedigree method would be more successful when resistance is governed by a major gene, while it does not give much improvements in combining minor genes in the population. While, single back cross-selected bulk breeding approach (Singh and Trethowan, 2007) can be used to transfer multiple minor resistance genes into a well-adapted and higher-yielding backgrounds (Singh et al., 2014). We have enlisted a few of popular Indian wheat varieties developed through conventional introgression of resistance genes for all three kinds of rusts (**Table 3**).

**Table 3:** List of popular Indian wheat varieties and their known rust resistant source.

Variety	Pedigree	Gene combination (s)		
		Leaf rust *(Lr)*	Stripe rust *(Yr)*	Stem rust *(Sr)*
LOK 1	S308/S331	*Lr13*	*Yr2ks*	*Sr2+Sr9b+Sr11*
PBW502	WH485/PBW343//RAJ1482	*Lr26+*	*Yr9+*	*Sr2+ Sr5+Sr31*
PBW343	ND/VG9144//KAL/BB/3/YCO”S”/4/VEE#S “S”	*Lr26+*	*Yr9+Yr27*	*Sr2+ Sr5+Sr31*
GW273	CPAN2084/VW205	*Lr13+Lr10*	*-*	*Sr8b+Sr11*
GW322	PBW173/GW196	*Lr13*	*-*	*Sr2+Sr11*
RAJ3765	HD2402/VL639	*Lr13+Lr10*	*Yr2ks+*	*Sr2+*
WH711	ALD’S’HUAC//HD2285/3/HFW-17	*Lr13*	*Yr2ks*	*Sr2+Sr7b*
DBW17	CMH79A.95/3*CNO79//RAJ3777	*Lr26+Lr23*	*Yr9+*	*Sr31*
PBW550	WH 594/RAJ 3856//W 485	*Lr26+R*	*Yr9+Yr18*	*Sr31*
PBW373	ND/VG9144//KAL/BB/3/YCO”S”/4/VEE#S “S”	*Lr26+*	*Yr9+Yr27*	*Sr2+ Sr5+Sr31*
RAJ4037	DL788-2/RAJ3717	*Lr24*	*-*	*Sr2+Sr24*
HD2189	HD2963/HD1931	*Lr13+Lr34*	*Sr2+Sr11*	*Yr2+Yr18*
WH147	E4870/C303//S339/PV18	*Lr13+Lr34*	*Yr2ks+Yr18*	*Sr7a+Sr11*
UP2338	UP368/VL421//UP262	*Lr26+Lr34*	*Yr9+Yr18*	*Sr2+Sr31*
WH542	JUPATECO/BLUE JAY//URES	*Lr26+Lr23*	*Yr9+Yr18*	*Sr31*
HUW234	HUW12*2/CPAN1666//HUW12	*Lr14a*	*Yr2ks+*	*Sr9b+Sr11*
HD2329	HD1962/E4870/3/K65/3/HD1553/UP262	*Lr13+Lr10+Lr34*	*Yr2+Yr18*	*Sr8b+Sr9b+Sr11*
C306	REGENT1974/3*CHZ//*2C591/3/C217/N14/C281	*Lr34*	*Yr2ks+Yr18*	*-*
HD2285	HD1912/HD1592//HD1962/E4870/3/K65/4/HD2160/HD2186	*Lr23*	*Yr2+*	*Sr9b+Sr11*
SONALIKA	II53.388/AN//YT54/N10B/3/LR/4/B4946.A.4.18.2IY/Y53//3*Y50	*Lr13*	*Yr2+*	*Sr2+Sr11*
UP262	S308/BJ66	*Lr23+Lr34*	*Yr2+Yr18*	*Sr2+Sr11*
PBW226	WG138/JUSTIN//CHIRS/HD1941	*Lr23*	*Yr2+*	*Sr2+*
MACS2496	A selection from SERI ‘S’	*Lr26+Lr23+Lr1+*	*Yr9+*	*Sr2+Sr31*
WH283	HD1981/RAJ821	*Lr10+*	*Yr2+*	*Sr8b+*
GW190	VEE/3/BB ‘S’/SKA//ARJUN	*Lr26+Lr23+Lr1+*	*Yr9+*	*Sr2+Sr31*
HS 295	CQT/AZ//IAS55/ALD/3/ALD/NAFN/4/PJN/PEL SL 1276.69	*Lr23+Lr34*	*Yr2ks+Yr18*	*Sr2+Sr8b+Sr11*
HD2967	ALD/CUC//URES/HD2160M/HD2278	*Lr23+*	*Yr2+*	*Sr8a+Sr11+Sr2*
HD3086	DBW14/HD2733//HUW468	*Lr13+Lr10+*	*Yr2+*	*Sr7b+Sr2*
WH1105	MILAN/S87230//BABAX	*Lr13+*	*Yr2+*	*R+*

Nayar et al. (2001); Bhardwaj (2011); Singh et al. (2011).

### Genomics Assisted Improvement of Rust Resistance

Most of the breeding programs with rust resistance as a major focus, work on identification, mapping, isolation, and introgression of diverse resistant genes into agronomically potential but, rust susceptible wheat lines. Though conventional breeding methods are effective, they are very time consuming, need laborious efforts to develop improved versions of crop plants for any target trait/s. In the last two decades, several genomic interventions have become possible which provide tremendous capabilities to characterize and manipulate wheat genome and to accelerate breeding. Though rust resistance has clear-cut phenotype to select for, the advent of molecular marker systems like SNPs and high-throughput genotyping platforms like SNP arrays have enabled precise estimation of marker-trait associations and hence, to select for the trait of interest without the greater involvement of environmental effects and to combine multiple genes at the same time.

To identify and map novel rust resistance genes, the biparental and genome-wide association based mapping are most widely followed, with the latter having the ability to localize multiple genes and/or QTLs. Rust resistance based on a single dominant gene is generally considered to be leading to genetic changes in pathogen virulence (Wilcoxon, 1981) and when incorporated singly into cultivars, it may become ineffective within a few years of cultivation. Hence, it is always recommended to use combinations of major and minor genes (Roelfs et al., 1992) which could confer broad-spectrum resistance. Here, MAS may be incorporated into backcross breeding to simultaneously introgress several rust resistant genes against a wider range of pathogens. Rapid generation advance protocols like doubled haploids (DH) and Speed breeding combined with marker-assisted breeding, would also assist in developing resistant cultivars in a very short period. Although MAS is a very efficient breeding strategy, its implementation is limited due to unavailability of tightly liked markers and can only identify few large effect QTLs.

In recent years, GS, an advanced version of MAS, is being largely used to increase the selection efficiency and to dissect complex polygenic traits with lower heritability. GS has the power to capture small individual effects of thousands of genes through whole genome prediction models, to estimate breeding values for the complex traits and hence overcome the problems associated with the MAS. Breeding for race nonspecific minor gene resistance is one way to minimize genotype and environment interaction of resistance, which in turn leads to stability in resistance and yield (Bekele et al., 2019). Breeders have already preferred to go for many minor gene based resistance instead of major genes, where GS is considered to be the best molecular breeding approach in place of MAS. The integration of GS with selected bulk, recurrent selection, and back cross breeding is being explored to maximize the genetic gain for quantitative traits like APR through selection. Transgene-free genetic engineering through genome editing with sequence specific nucleases has paved a way for manipulating any specific genomic sequence superseding random mutagenesis (Wang et al., 2018). Among the four types of nucleases used in genome editing, CRISPR/Cas9 has a higher success rate in gene modification and requires less know-how. The application of genome editing for disease resistance would be much feasible; due to the better understanding of the molecular mechanisms underlying resistance, modification of a single gene may lead to resistance phenotype, and targeted mutagenesis is well applicable to knockout susceptible genes (Borrelli et al., 2018).

### Deployment of Adult Plant Genes Delaying the Evolution of Pathotypes

Race-specific APRs which can be screened at the greenhouse level at two or third-leaf stage like *Lr13* and *Yr49* (McIntosh et al., 1995) are more vulnerable to succumb matching virulence in rust pathogens. Race-specific APR *Lr13* expresses at the seedling stage (two-leaf stage) in a greenhouse as well as at field conditions against avirulent pathotypes. Due to their expression at an early stage, some researchers have cited these genes as seedling resistance genes (Chen and Kang, 2017). APR *Lr22a* confers leaf rust resistance under field conditions and rendered resistance against 22 leaf rust pathotypes at flag leaf stage (Sawhney et al., 1982). *Lr34* was reported to be partially effective against leaf rust pathotypes in India (Sawhney and Sharma, 1990) and it expresses enhanced resistance along with other resistance genes (German and Kolmer, 1992). APR *Lr34* carrying genotype reduced the yield losses up to 18% and genotype lacking *Lr34* revealed 60 to 84% of losses (Singh et al., 1994). Slow rusting characters of *Lr34* and its interaction with other genes have made it useful for rust resistance (Nayar et al., 1999). Similarly, *Lr46/Yr29/Pm39* gene complex was identified in “Pavon76” and conferred a slow rusting to leaf rust, stripe rusts, and powdery mildew in wheat (William et al., 2003; Lillemo et al., 2008; Singh et al., 2013). The leaf rust resistance genes *Lr48* and *Lr49* were reported in wheat cultivars CSP44 and VL404, respectively as hypersensitive adult plant resistance genes (Saini et al., 2002). Another gene *Lr67/Yr46/Sr55* also confers slow rusting to leaf and stripe rusts (Hiebert et al., 2010; Herrera-Foessel et al., 2011). An Australian Pst pathotype 239 E237 A- 17 + 33+ has changed the rust rating of Australian cultivar “Axe” carrying *Yr75* (Cuddy and Hollway, 2018). Previous studies showed the susceptible nature of *Yr49* against Chinese Pst races (Ellis et al., 2014). Several stripe rust APRs have been overcome by newly detected Pst races in Europe and North America, so those APRs were tentatively classified as race-specific APRs (Hao et al., 2011; Sthapit et al., 2012; Sørensen et al., 2014). An accession HS628 expressed broad-spectrum resistance to Pt, Pst, and Pgt pathotypes in India (Pal et al., 2018). Developing wheat varieties possessing these diverse gene combinations could be a feasible approach to delay the evolution of pathotypes with matching virulence genes.

## Concluding Remarks

Breeding for rust resistance is an integral part of wheat improvement. APRs *Sr2*, *Lr34*, *Lr46*, *Lr67*, and *Lr68* have been found durable and non-race specific. These APRs can offer only partial resistance which is insufficient to address food insecurity threat. Deployment of both durable rust resistance genes along with major R genes has been reported as a sound breeding strategy to avoid rust epidemics worldwide. Development of gene-specific DNA markers is essential to introgress the rust resistance gene in the desired wheat background and avoid linkage drag. Positional-cloning and MutRenSeq approach along with available genomics resources were explored to develop gene-specific marker of around twenty rust resistance genes including four APRs (*Lr34*, *Lr67*, *Yr36* and *Lr22a*). Stacking of five cloned resistance genes in modern wheat variety is underway *via* cis-genic approach. In future, this approach will be fruitful to develop resistant wheat against three rusts. Rust pathogen has been continuously evolving to shrink the number of effective rust resistance genes. The high-quality reference genome of over ten wheat varieties (10+ genome project) will assist in the cloning of additional major genes and APR. The major future challenge would be to identify effective resistance genes and their deployment to attain durable rust resistance.

## Author Contributions

PB, DB, DW, HB, and AS wrote the manuscript. All authors contributed to the article edits and approved the submitted version.

## Conflict of Interest

The authors declare that the research was conducted in the absence of any commercial or financial relationships that could be construed as a potential conflict of interest.

## References

[B1] Abe A. Kosugi S. Yoshida K. Natsume S. Takagi H. Kanzaki H. (2012). Genome sequencing reveals agronomically important loci in rice using MutMap. Nat. Biotechnol. 30, 174. 10.1038/nbt.2095 22267009

[B2] Aboukhaddour R. Fetch T. McCallum B. D. Harding M. W. Beres B. L. Graf R. J. (2020). Wheat diseases on the prairies: A Canadian story. Plant Pathol. 69 (3), 418–432. 10.1111/ppa.13147

[B3] Aggarwal R. Kulshreshtha D. Sharma S. Singh V. K. Manjunatha C. Bhardwaj S. C. (2018). Molecular characterization of Indian pathotypes of *Puccinia striiformis* f. sp. *tritici* and multigene phylogenetic analysis to establish inter- and intraspecific relationships. Genet. Mol. Biol. 41 (4), 834–842. 10.1590/1678-4685-GMB-2017-0171 30281059 PMC6415613

[B4] Aime M. C. Bell C. D. Wilson A. W. (2018). Deconstructing the evolutionary complexity between rust fungi (Pucciniales) and their plant hosts. Stud. Mycol. 89, 143–152. 10.1016/j.simyco.2018.02.002 29910520 PMC6002339

[B5] Anderson J. Ogihara Y. Sorrells M. Tanksley S. (1992). Development of a chromosomal arm map for wheat based on RFLP markers. Theor. Appl. Genet. 83 (8), 1035–1043. 10.1007/BF00232969 24202932

[B6] Anderson R. G. (1963). Studies on the inheritance of resistance to leaf rust of wheat. Proc. 2nd Int. Wheat Genet. Symp. Lund, 144–155.

[B7] Appels R. Eversole K. Stein N. Feuillet C. Keller B. Rogers J. (2018). Shifting the limits in wheat research and breeding using a fully annotated reference genome. Science 361 (6403), eaar7191. 10.1126/science.aar7191 30115783

[B8] Arora S. Steuernagel B. Chandramohan S. Long Y. Matny O. Johnson R. (2018). Resistance gene discovery and cloning by sequence capture and association genetics. bioRxiv 248146. 10.1038/s41587-018-0007-9 30718880

[B9] Asad M. A. Xia X. Wang C. He Z. (2012). Molecular mapping of stripe rust resistance gene *YrSN104* in Chinese wheat line Shaannong 104. Hereditas 149 (4), 146–152. 10.1111/j.1601-5223.2012.02261.x 22967144

[B10] Bansal U. Bariana H. (2017). ““Advances in Identification and Mapping of Rust Resistance Genes in Wheat”,” in Wheat Rust Diseases: Methods in Molecular Biology. Ed. Periyannan S. (New York, NY: Humana Press), 1659. 10.1007/978-1-4939-7249-4_13 28856648

[B11] Bansal U. Hayden M. J. Venkata B. P. Khanna R. Saini R. G. Bariana H. S. (2008). Genetic mapping of adult plant leaf rust resistance genes *Lr48* and *Lr49* in common wheat. Theor. Appl. Genet. 17, 307–312. 10.1007/s00122-008-0775-6 18542911

[B12] Bansal U. Bariana H. Wong D. Randhawa M. Wicker T. Hayden M. (2014). Molecular mapping of an adult plant stem rust resistance gene Sr56 in winter wheat cultivar Arina. Theor. Appl. Genet. 127 (6), 1441–1448. 10.1007/s00122-014-2311-1 24794977

[B13] Bansal M. Kaur S. Dhaliwal H. S. Bains N. S. Bariana H. S. Chhuneja P. (2017). Mapping of *Aegilops umbellulata*-derived leaf rust and stripe rust resistance loci in wheat. Plant Pathol. 66 (1), 38–44. 10.1111/ppa.12549

[B14] Bariana H. S. Hayden M. J. Ahmed N. Bell J. Sharp P. McIntosh R. (2001). Mapping of durable adult plant and seedling resistances to stripe rust and stem rust diseases in wheat. Crop Pasture Sci. 52 (12), 1247–1255. 10.1071/AR01040

[B15] Bariana H. S. Brown G. N. Bansal U. K. Miah H. Standen G. E. Lu M. (2007). Breeding triple rust resistant wheat cultivars for Australia using conventional and marker assisted selection technologies. Aust. J. Agric. Res. 58 (6), 576. 10.1071/ar07124

[B16] Bariana H. S. Bansal U. K. Basandrai D. Chhetri M. (2013). “Disease Resistance,” in Genomics and Breeding for Climate-Resilient Crops, vol 2 Target Traits. Ed. Kole C. (Berlin: Springer-Verlag Berlin Heidelberg), 291–314.

[B17] Bariana H. Forrest K. Qureshi N. Miah H. Hayden M. Bansal U. (2016). Adult plant stripe rust resistance gene *Yr71* maps close to *Lr24 in* chromosome 3D of common wheat. Mol. Breed. 36 (7), 1–10. 10.1007/s11032-016-0528-1

[B18] Basnet B. R. Singh R. P. Ibrahim A. M. H. Herrera-Foessel S. A. Huerta-Espino J. Lan C. (2014). Characterization of *Yr54* and other genes associated with adult plant resistance to yellow rust and leaf rust in common wheat Quaiu 3. Mol. Breed. 33 (2), 385–399. 10.1007/s11032-013-9957-2

[B19] Bekele D. Tesfaye K. Fikre A. (2019). Recent Developments in Genomic Selection for Minor Gene Quantitative Disease Resistance Plant Breeding. J. Plant Pathol. Microbiol. 10 (478), 1–8. 10.35248/2157-7471.10.478

[B20] Beresford R. M. (1982). Stripe rust (*Puccinia striiformis*), a new disease of wheat in New Zealand. Cer. Rusts Bull. 10, 35–41.

[B21] Bhardwaj S. C. (2011). "Resistance genes and adult plant rust resistance of released wheat varieties of India", in Research Bulletin 5. (Flowerdale, Shimla, India: Directorate of Wheat Research, Regional Station), 31.

[B22] Bhardwaj S. C. Singh G. P. Gangwar O. P. Prasad P. Kumar S. (2019). Status of Wheat Rust Research and Progress in Rust Management-Indian Context. Agronomy 9 (12) 1–14. 10.3390/agronomy9120892

[B23] Bhavani S. Hodson D. P. Huerta-espino J. Randhawa M. S. Singh R. P. (2019). Progress in breeding for resistance to Ug99 and other races of the stem rust fungus in CIMMYT wheat germplasm. Front. Agr. Sci. Eng. 6 (3), 210–224. 10.15302/J-FASE-2019268

[B24] Biffen R. H. (1905). Mendel’s laws of inheritance and wheat breeding. J. Agric. Sci. 1, 14–48. 10.1017/S0021859600000137

[B25] Borrelli V. M. G. Brambilla V. Rogowsky P. Marocco A. Lanubile A. (2018). The Enhancement of Plant Disease Resistance Using CRISPR/Cas9 Technology. Front. Plant Sci. 9, 1245. 10.3389/fpls.2018.01245 30197654 PMC6117396

[B26] Borrill P. Adamski N. Uauy C. (2015). Genomics as the key to unlocking the polyploid potential of wheat. New Phytol. 208, 1008– 1022. 10.1111/nph.13533 26108556

[B27] Bradbury P. J. Zhang Z. Kroon D. E. Casstevens T. M. Ramdoss Y. Buckler E. S. (2007). TASSEL: software for association mapping of complex traits in diverse samples. Bioinformatics 23 (19), 2633–2635. 10.1093/bioinformatics/btm308 17586829

[B28] Brenchley R. Spannagl M. Pfeifer M. Barker G. L. D’Amore R. Allen A. M. (2012). Analysis of the bread wheat genome using whole-genome shotgun sequencing. Nature 491, 705–710. 10.1038/nature11650 23192148 PMC3510651

[B29] Cavanagh C. R. Chao S. Wang S. Huang B. E. Stephen S. Kiani S. (2013). Genome-wide comparative diversity uncovers multiple targets of selection for improvement in hexaploid wheat landraces and cultivars. Proc. Natl. Acad. Sci. U. S. A. 110 (20), 8057–8062. 10.1073/pnas.1217133110 23630259 PMC3657823

[B30] Chapman J. A. Mascher M. Buluç A. Barry K. Georganas E. Session A. (2015). A whole-genome shotgun approach for assembling and anchoring the hexaploid bread wheat genome. Genome Biol. 16 (26), 1–17. 10.1186/s13059-015-0582-8 25637298 PMC4373400

[B31] Chaves M. S. Martinelli J. A. Wesp-Guterres C. Graichen F. A. S. Brammer S. Scagliusi S. M. (2013). The importance for food security of maintaining rust resistance in wheat. Food Secur. 5 (2), 157–176. 10.1007/s12571-013-0248-x

[B32] Chen X. Kang Z. (2017). “Introduction: history of research, symptoms, taxonomy of the pathogen, host range, distribution, and impact of stripe rust,” in Stripe Rust. Eds. Chen X. Kang Z. (Dordrect, Netherlands: Springer Science), 1–33.

[B33] Chen S. Zhang W. Bolus S. Rouse M. N. Dubcovsky J. (2018). Identification and characterization of wheat stem rust resistance gene *Sr21* effective against the Ug99 race group at high temperature. PloS Genet. 14, e1007287. 10.1371/journal.pgen.1007287 29614079 PMC5882135

[B34] Chen C. Clark B. Martin M. J. Matny O. N. Steffenson B. J. Franckowiak J. D. (2020). Ancient BED-domain-containing immune receptor from wild barley confers widely effective resistance to leaf rust. bioRxiv. 10.1101/2020.01.19.911446 PMC8196641 33415836

[B35] Chen S. Rouse M. N. Zhang W. Zhang X. Guo Y. Briggs J. (2020). Wheat gene *Sr60* encodes a protein with two putative kinase domains that confers resistance to stem rust. New Phytol. 225 (2), 948–959. 10.1111/nph.16169 31487050

[B36] Chen X. (2005). Epidemiology and control of stripe rust [*Puccinia striiformis* f. sp. *tritici*] on wheat. Can. J. Plant Pathol. 27 (3), 314–337. 10.1080/07060660509507230

[B37] Chhetri M. Bariana H. Wong D. Sohail Y. Hayden M. Bansal U. (2017). Development of robust molecular markers for marker-assisted selection of leaf rust resistance gene *Lr23 in* common and durum wheat breeding programs. Mol. Breed. 37 (3), 21. 10.1007/s11032-017-0628-6

[B38] Cloutier S. McCallum B. D. Loutre C. Banks T. W. Wicker T. Feuillet C. (2007). Leaf rust resistance gene *Lr1*, isolated from bread wheat (*Triticum aestivum* L.) is a member of the large psr567 gene family. Plant Mol. Biol. 65 (1), 93–106. 10.1007/s11103-007-9201-8 17611798

[B39] Collard B. C. Y. Jahufer M. Z. Z. Brouwer J. B. Pang E. C. K. (2005). An introduction to markers, quantitative trait loci (QTL) mapping and marker-assisted selection for crop improvement: The basic concepts. Euphytica 142 (1-2), 169–196. 10.1007/s10681-005-1681-5

[B40] Cuddy W. Hollway G. (2018). Detection of a new wheat stripe rust pathotype in Victoria. Cereal Rust Rep. 16 (1), 1.

[B41] Davey J. W. Hohenlohe P. A. Etter P. D. Boone J. Q. Catchen J. M. Blaxter M. L. (2011). Genome-wide genetic marker discovery and genotyping using next-generation sequencing. Nat. Rev. Genet. 12 (7), 499–510. 10.1038/nrg3012 21681211

[B42] Dinh H. X. Singh D. Periyannan S. Park R. F. Pourkheirandish M. (2020). Molecular genetics of leaf rust resistance in wheat and barley. Theor. Appl. Genet. 133, 2035–2050. 10.1007/s00122-020-03570-8 32128617

[B43] Dodds P. Lawrence G. Mago R. Ayliffe M. Narayana U. Szabo L. (2010). “Advances in host-pathogen molecular interactions: rust effectors as targets for recognition,” in Oral papers 2009 Technical Workshop (Full Papers and Abstracts for the Presented Talks), (Obregón, Sonora, Mexico: Borlaug Global Rust Initiative, Cd.) 49–54.

[B44] Dong Z. Hegarty J. M. Zhang J. Zhang W. Chao S. Chen X. (2017). Validation and characterization of a QTL for adult plant resistance to stripe rust on wheat chromosome arm 6BS (*Yr78*). Theor. Appl. Genet. 130 (10), 2127–2137. 10.1007/s00122-017-2946-9 28725946 PMC5606939

[B45] Dracatos P. M. Zhang P. Park R. F. McIntosh R. A. Wellings C. R. (2016). Complementary resistance genes in wheat selection ‘Avocet R’ confer resistance to stripe rust. Theor. Appl. Genet. 129 (1), 65–76. 10.1007/s00122-015-2609-7 26433828

[B46] Dracatos P. M. Bartos J. Elmansour H. Singh D. Karafiatova M. Zhang P (2019). The coiled-coil NLR *Rph1*, confers leaf rust resistance in barley cultivar Sudan. Plant Physiol. 179, 1362. 10.1104/pp.18.01052 30593453 PMC6446784

[B47] Drenkard E. Richter B. G. Rozen S. Stutius L. M. Angell N. A. Mindrinos M. (2000). A simple procedure for the analysis of single nucleotide polymorphisms facilitates map-based cloning in Arabidopsis. Plant Physiol. 124 (4), 1483–1492. 10.1104/pp.124.4.1483 11115864 PMC1539302

[B48] Dubin H. J. Torres E. (1981). Causes and consequences of the 1976-1977 wheat leaf rust epidemic in northwest Mexico. Annu. Rev. Phytopathol. 19, 41–49. 10.1146/annurev.py.19.090181.000353

[B49] Duveiller E. Singh R. P. Nicol J. M. (2007). The challenges of maintaining wheat productivity: pests, diseases, and potential epidemics. Euphytica 157, 417–430. 10.1007/s10681-007-9380-z

[B50] El Baidouri M. Murat F. Veyssiere M. Molinier M. Flores R. Burlot L. (2017). Reconciling the evolutionary origin of bread wheat (*Triticum aestivum*). New Phytol. 213 (3), 1477–1486. 10.1111/nph.14113 27551821

[B51] Eliana D. Brent G. R. Steve R. Lisa M. S. Nathaniel A. A. Michael M. (2002). A Simple Procedure for the Analysis of Single Nucleotide Polymorphisms Facilitates Map-Based Cloning in Arabidopsis1. Plant Physiol. 124 (4), 1483–1492. 10.1104/pp.124.4.1483 PMC1539302 11115864

[B52] Ellis J. G. Lagudah E. S. Spielmeyer W. Dodds P. N. (2014). The past, present and future of breeding rust resistant wheat. Front. Plant Sci. 5, 641. 10.3389/fpls.2014.00641 25505474 PMC4241819

[B53] FAOSTAT (2020). Statistical databases. Food and Agriculture Organization of the United Nations. Available at: http://www.fao.org/faostat/en/#home (Accessed on 31 March 2020).

[B54] Feldman M. (1995). “Wheats,” in Evolution of crop plants. Eds. Smartt J. Simmonds N. W. (Harlow, UK: Longman Scientific and Technical), 185–192.

[B55] Feng J. Wang M. See D. R. Chao S. Zheng Y. Chen X. (2018). Characterization of novel gene *Yr79* and four additional quantitative trait loci for all-stage and high-temperature adult-plant resistance to stripe rust in spring wheat PI 182103. Phytopathology 108 (6), 737–747. 10.1094/Phyto-11-17-0375-r 29303685

[B56] Feuillet C. Travella S. Stein N. Albar L. Nublat A. Keller B. (2003). Map-based isolation of the leaf rust disease resistance gene *Lr10* from the hexaploid wheat (*Triticum aestivum* L.) genome. Proc. Natl. Acad. Sci. U. S. A. 100 (25), 15253–15258. 10.1073/pnas.2435133100 14645721 PMC299976

[B57] Figueroa M. Upadhyaya N. M. Sperschneider J. Park R. F. Szabo L. J. Steffenson B. (2016). Changing the game: using integrative genomics to probe virulence mechanisms of the stem rust pathogen Puccinia graminis f. sp. tritici. Front. Plant Sci. 7, 205. 10.3389/fpls.2016.00205 26941766 PMC4764693

[B58] Flor H. H. (1956). The complementary genic system in flax and flax rust. Adv. Genet. 8, 29–54. 10.1016/S0065-2660(08)60498-8

[B59] Fu D. Uauy C. Distelfeld A. Blechl A. Epstein L. Chen X. (2009). A kinase-START gene confers temperature-dependent resistance to wheat stripe rust. Science 323 (5919), 1357–1360. 10.1126/science.1166289 19228999 PMC4737487

[B60] Fudal I. Ross S. Gout L. Blaise F. Kuhn M. L. Eckert M. R. (2007). Heterochromatin-like regions as ecological niches for avirulence genes in the leptosphaeria maculans genome: map-based cloning of AvrLm6. Mol. Plant Microbe Interact. 20 (4), 459–470. 10.1094/MPMI-20-4-0459 17427816

[B61] Gao L. Kielsmeier-Cook J. Bajgain P. Zhang X. Chao S. Rouse M. N. (2015). Development of genotyping by sequencing (GBS)-and array-derived SNP markers for stem rust resistance gene Sr42. Mol. Breed. 35 (207), 2–13. 10.1007/s11032-015-0404-4

[B62] German S. E. Kolmer J. A. (1992). Effect of gene *Lr34* in the enhancement of resistance to leaf rust of wheat. Theor. Appl. Genet. 84 (1), 97–105. 10.1007/bf00223987 24203034

[B63] Gessese M. Bariana H. Wong D. Hayden M. Bansal U. (2019). Molecular mapping of stripe rust resistance gene *Yr81* in a common wheat landrace Aus27430. Plant Dis. 103, 1166–1171. 10.1094/PDIS-06-18-1055-RE 30998448

[B64] Ghazvini H. Hiebert C. W. Thomas J. B. Fetch T. (2012). Development of a multiple bulked segregant analysis (MBSA) method used to locate a new stem rust resistance gene (*Sr54*) in the winter wheat cultivar Norin 40. Theor. Appl. Genet. 126 (2), 443–449. 10.1007/s00122-012-1992-6 23052026

[B65] Gill U. S. Lee S. Mysore K. S. (2015). Host versus non-host resistance: distinct wars with similar arsenals. Phytopathology 105 580, 587. 10.1094/phyto-11-14-0298-rvw 25626072

[B66] Hao Y. Chen Z. Wang Y. Bland D. Buck J. Brown-Guedira G. (2011). Characterization of a major QTL for adult plant resistance to stripe rust in US soft red winter wheat. Theor. Appl. Genet. 123 (8), 1401–1411. 10.1007/s00122-011-1675-8 21830107

[B67] Hayes H. K. Pafker J. H. Kurtzweil C. (1920). Genetics of rust resistance in crosses of varieties of *Triticum vulgare* with varieties of *T. durum* and *T dicoccum* . J. Agric. Res. 19, 523–542.

[B68] Hayes H. K. Ausemus E. R. Stakman E. C. Bailey C. H. Wilson H. K. Bamberg R. H. (1936). Thatcher wheat. S. Bull. Minn. Agric. Exp. Stn. 325, 39.

[B69] Helguera M. Khan I. A. Kolmer J. Lijavetzky D. Zhong-qi L. Dubcovsky J. (2003). PCR assays for the *Lr37-Yr17-Sr38* cluster of rust resistance genes and their use to develop isogenic hard red spring wheat lines. Crop Sci. 43 (5) 1839–1847. 10.2135/cropsci2003.1839

[B70] Herrera-Foessel S. A. Lagudah E. S. Huerta-Espino J. Hayden M. J. Bariana H. S. Singh D. (2011). New slow-rusting leaf rust and stripe rust resistance genes *Lr67* and *Yr46* in wheat are pleiotropic or closely linked. Theor. Appl. Genet. 122 (1), 239–249. 10.1007/s00122-010-1439-x 20848270

[B71] Herrera-Foessel S. A. Singh R. P. Huerta-Espino J. Rosewarne G. M. Periyannan S. K. Viccars L. (2012). *Lr68*: a new gene conferring slow rusting resistance to leafrust in wheat. Theor. Appl. Genet. 124, 1475–1486. 10.1007/s00122-012-1802-1 22297565

[B72] Herrera-Foessel S. A. Huerta-Espino J. Calvo-Salazar V. Lan C. X. Singh R. P. (2013). *Lr72* confers resistance to leaf rust in durum wheat cultivar Atil C2000. Plant Dis. 98 (5), 631–635. 10.1094/PDIS-07-13-0741-RE 30708548

[B73] Herrera-Foessel S. A. Singh R. P. Lan C. X. Huerta-Espino J. Calvo-Salazar V. Bansal U. K. (2014a). *Yr60*, a gene conferring moderate resistance to stripe rust in wheat. Plant Dis. 99 (4), 508–511. 10.1094/PDIS-08-14-0796-RE 30699549

[B74] Herrera-Foessel S. A. Singh R. P. Lillemo M. Huerta-Espino J. Bhavani S. Singh S. (2014b). *Lr67/Yr46* confers adult plant resistance to stem rust and powdery mildew in wheat. Theor. Appl. Genet. 127 (4), 781–789. 10.1007/s00122-013-2256-9 24408377

[B75] Hiebert C. W. Thomas J. B. McCallum B. D. Humphreys D. G. DePauw R. M. Hayden M. J. (2010). An introgression on wheat chromosome 4DL in RL6077 (Thatcher*6/PI 250413) confers adult plant resistance to stripe rust and leaf rust (*Lr67*). Theor. Appl. Genet. 121, 1083–1091. 10.1007/s00122-010-1373-y 20552325

[B76] Huang L. Brooks S. A. Li W. Fellers J. P. Trick H. N. Gill B. S. (2003). Map-based cloning of leaf rust resistance gene *Lr21* from the large and polyploid genome of bread wheat. Genetics 164 (2), 655–664. 12807786 10.1093/genetics/164.2.655 PMC1462593

[B77] Huerta-Espino J. Singh R. Germán S. McCallum B. Park R. Chen W. (2011). Global status of wheat leaf rust caused by *Puccinia triticina* . Euphytica 179 (1), 143–160. 10.1007/s10681-011-0361-x

[B78] Hussain M. Hassan S. F. Kirmani M. A. S. (1980). “Virulence in Puccinia recondita Rob. ex. Desm. f. sp. tritici in Pakistan during 1978 and 1979,” in Proceedings of 5th European and Mediterranean cereal rust conference(Bari, Italy), 179–184.

[B79] International Wheat Genome Sequencing, C (2014). A chromosome-based draft sequence of the hexaploid bread wheat (*Triticum aestivum*) genome. Science 345 (6194), 1251788. 10.1126/science.1251788 25035500

[B80] Jander G. Norris S. R. Rounsley S. D. Bush D. F. Levin I. M. Last R. L. (2002). Arabidopsis map-based cloning in the post-genome era. Plant Physiol. 129 (2), 440–450. 10.1104/pp.003533 12068090 PMC1540230

[B81] Jin Y. Szabo L. J. Carson M. (2010). Century - old mystery of *Puccinia striiformis* life history solved with the identification of *Berberis* as an alternate host. Phytopathology 100, 432–435. 10.1094/PHYTO-100-5-0432 20373963

[B82] Johnson R. Law C. N. (1975). Genetic control of durable resistance to yellow rust (*Puccinia striiformis*) in the wheat cultivar Hybrid de Bersee. Ann. Appl. Biol. 81, 385. 10.1111/j.1744-7348.1975.tb01654.x

[B83] Joshi L. M. Srivastava K. D. Nagarajan S. (1975). An analysis of brown rust epidemics of 1971-72 and 1972-73. Indian Phytopathol. 28, 138.

[B84] Juliana P. Rutkoski J. E. Poland J. A. Singh R. P. Murugasamy S. Natesan S. (2015). Genome-Wide Association Mapping for Leaf Tip Necrosis and Pseudo-black Chaff in Relation to Durable Rust Resistance in Wheat. Plant Genome 8 (2), 1–12. 10.3835/plantgenome2015.01.0002 33228313

[B85] Juliana P. Singh R. P. Singh P. K. Poland J. A. Bergstrom G. C. Huerta-Espino J. (2018). Genome-wide association mapping for resistance to leaf rust, stripe rust and tan spot in wheat reveals potential candidate genes. Theor. Appl. Genet. 131 (7), 1405–1422. 10.1007/s00122-018-3086-6 29589041 PMC6004277

[B86] Jupe F. Witek K. Verweij W. Sliwka J. Pritchard L. Etherington G. J. (2013). Resistance gene enrichment sequencing (RenSeq) enables reannotation of the NB-LRR gene family from sequenced plant genomes and rapid mapping of resistance loci in segregating populations. Plant J. 76 (3), 530–544. 10.1111/tpj.12307 23937694 PMC3935411

[B87] Kassa M. T. You F. M. Hiebert C. W. Pozniak C. J. Fobert P. R. Sharpe A. G. (2017). Highly predictive SNP markers for efficient selection of the wheat leaf rust resistance gene *Lr16* . BMC Plant Biol. 17 (1), 45. 10.1186/s12870-017-0993-7 28202046 PMC5311853

[B88] Keeble-Gagnère G. Isdale D. Suchecki R. Kruger A. Lomas K. Carroll D. (2019). Integrating past, present and future wheat research with Pretzel. bioRxiv 4 1–4. 10.1101/517953

[B89] Kihara H. (1944). Discovery of the DD analyser, one of the ancestors of T. vulgare. Agric. Hortic. 19, 13–14.

[B90] Kilian A. Chen J. Han F. Steffenson B. Kleinhofs A. (1997). Towards map-based cloning of the barley stem rust resistance genes *Rpg1* and *Rpg4* using rice as an intergenomic cloning vehicle. Plant Mol. Biol. 35 (1/2), 187–195. 10.1023/a:1005768222615 9291972

[B91] Klymiuk V. Yaniv E. Huang L. Raats D. Fatiukha A. Chen S. (2018). Cloning of the wheat *Yr15* resistance gene sheds light on the plant tandem kinase-pseudokinase family. Nat. Commun. 9 (1), 3735. 10.1038/s41467-018-06138-9 30282993 PMC6170490

[B92] Kolmer J. Chen X. Jin Y. (2009). “Diseases which challenge global wheat production-The wheat rusts,” in Wheat Science and Trade. Ed. Carver B. F. (USA: Wiley-Blackwell), 89–124.

[B93] Komori T. Ohta S. Murai N. Takakura Y. Kuraya Y. Suzuki S. (2004). Map-based cloning of a fertility restorer gene, *Rf-1*, in rice (*Oryza sativa* L.). Plant J. 37 (3), 315–325. 10.1046/j.1365-313x.2003.01961.x 14731253

[B94] Krattinger S. G. Lagudah E. S. Spielmeyer W. Singh R. P. Huerta-Espino J. McFadden H. (2009). A putative ABC transporter confers durable resistance to multiple fungal pathogens in wheat. Science 323 (5919), 1360–1363. 19229000 10.1126/science.1166453

[B95] Krattinger S. G. Lagudah E. S. Wicker T. Risk J. M. Ashton A. R. Selter L. L. (2011). *Lr34* multi-pathogen resistance ABC transporter: molecular analysis of homoeologous and orthologous genes in hexaploid wheat and other grass species. Plant J. 65 (3), 392–403. 10.1111/j.1365-313X.2010.04430.x 21265893

[B96] Krattinger S. G. Sucher J. Selter L. L. Chauhan H. Zhou B. Tang M. (2016). The wheat durable, multipathogen resistance gene *Lr34* confers partial blast resistance in rice. Plant Biotechnol. J. 14 (5), 1261–1268. 10.1111/pbi.12491 26471973 PMC11388880

[B97] Kthiri D. Loladze A. MacLachlan P. R. N’Diaye A. Walkowiak S. Nilsen K. (2018). Characterization and mapping of leaf rust resistance in four durum wheat cultivars. PloS One 13 (5), e0197317. 10.1371/journal.pone.0197317 29746580 PMC5945016

[B98] Lagudah E. S. McFadden H. Singh R. P. Huerta-Espino J. Bariana H. S. Spielmeyer W. (2006). Molecular genetic characterization of the *Lr34/Yr18* slow rusting resistance gene region in wheat. Theor. Appl. Genet. 114 (1), 21–30. 10.1007/s00122-006-0406-z 17008991

[B99] Lagudah E. S. Krattinger S. G. Herrera-Foessel S. Singh R. P. Huerta-Espino J. Spielmeyer W. (2009). Gene-specific markers for the wheat gene *Lr34/Yr18/Pm38* which confers resistance to multiple fungal pathogens. Theor. Appl. Genet. 119 (5), 889–898. 10.1007/s00122-009-1097-z 19578829

[B100] Li Z. Lan C. He Z. Singh R. P. Rosewarne G. M. Chen X. (2014). Overview and application of QTL for adult plant resistance to leaf rust and powdery mildew in wheat. Crop Sci. 54 (5), 1907–1925. 10.2135/cropsci2014.02.0162

[B101] Li H. Vikram P. Singh R. P. Kilian A. Carling J. Song J. (2015). A high density GBS map of bread wheat and its application for dissecting complex disease resistance traits. BMC Genomics 16 (1), 216. 10.1186/s12864-015-1424-5 25887001 PMC4381402

[B102] Li J. Yang J. Li Y. Ma L. (2020b). Current strategies and advances in wheat biology. Crop J. 10.1016/j.cj.2020.03.004

[B103] Li J. Dundas I. Dong C. Li G. Trethowan R. Yang Z. (2020a). Identification and characterization of a new stripe rust resistance gene *Yr83* on rye chromosome 6R in wheat. Theor. Appl. Genet. 133 (4), 1095–1107. 10.1007/s00122-020-03534-y 31955232

[B104] Lillemo M. Asalf B. Singh R. P. Huerta-Espino J. Chen X. M. He Z. H. (2008). The adult plant rust resistance loci *Lr34/Yr18* and *Lr46/Yr29* are important determinants of partial resistance to powdery mildew in bread wheat line Saar. Theor. Appl. Genet. 116, 1155–1166. 10.1007/s00122-008-0743-1 18347772

[B105] Lillemo M. Joshi A. K. Prasad R. Chand R. Singh R. P. (2013). QTL for spot blotch resistance in bread wheat line Saar co-locate to the biotrophic disease resistance loci *Lr34* and *Lr46* . Theor. Appl. Genet. 126 (3), 711–719. 10.1007/s00122-012-2012-6 23139144

[B106] Ling H. Q. Koch G. Baumlein H. Ganal M. W. (1999). Map-based cloning of chloronerva, a gene involved in iron uptake of higher plants encoding nicotianamine synthase. Proc. Natl. Acad. Sci. U. S. A. 96 (12), 7098–7103. 10.1073/pnas.96.12.7098 10359845 PMC22069

[B107] Ling H. Q. Zhu Y. Keller B. (2003). High-resolution mapping of the leaf rust disease resistance gene *Lr1* in wheat and characterization of BAC clones from the *Lr1* locus. Theor. Appl. Genet. 106 (5), 875–882. 10.1007/s00122-002-1139-2 12647062

[B108] Liu W. Rouse M. Friebe B. Jin Y. Gill B. Pumphrey M. O. (2011). Discovery and molecular mapping of a new gene conferring resistance to stem rust, *Sr53*, derived from *Aegilops geniculata* and characterization of spontaneous translocation stocks with reduced alien chromatin. Chromosome Res. 19 (5), 669–682. 10.1007/s10577-011-9226-3 21728140

[B109] Liu H. Bayer M. Druka A. Russell J. R. Hackett C. A. Poland J. (2014). An evaluation of genotyping by sequencing (GBS) to map the *Breviaristatum-e* (*ari-e*) locus in cultivated barley. BMC Genomics 15 (1), 1–11. 10.1186/1471-2164-15-104 24498911 PMC3922333

[B110] Liu W. Frick M. Huel R. Nykiforuk C. L. Wang X. Gaudet D. A. (2014). The stripe rust resistance gene *Yr10* encodes an evolutionary-conserved and unique CC-NBS-LRR sequence in wheat. Mol. Plant 7 (12), 1740–1755. 10.1093/mp/ssu112 25336565

[B111] Lowe I. Jankuloski L. Chao S. Chen X. See D. Dubcovsky J. (2011). Mapping and validation of QTL which confer partial resistance to broadly virulent post-2000 North American races of stripe rust in hexaploid wheat. Theor. Appl. Genet. 123 (1), 143–157. 10.1007/s00122-011-1573-0 21455722 PMC4761445

[B112] Lu Y. Wang M. Chen X. See D. Chao S. Jing J. (2014). Mapping of *Yr62* and a small-effect QTL for high-temperature adult-plant resistance to stripe rust in spring wheat PI 192252. Theor. Appl. Genet. 127 (6), 1449–1459. 10.1007/s00122-014-2312-0 24781075

[B113] Maccaferri M. Zhang J. Bulli P. Abate Z. Chao S. Cantu D. (2015). A genome-wide association study of resistance to stripe rust (*Puccinia striiformis* f. sp. *tritici*) in a worldwide collection of hexaploid spring wheat (*Triticum aestivum* L.). G3: Genes Genom. Genet. 5 (3), 449–465. 10.1534/g3.114.014563 PMC4349098 25609748

[B114] Mago R. Bariana H. S. Dundas I. S. Spielmeyer W. Lawrence G. J. Pryor A. J. (2005). Development of PCR markers for the selection of wheat stem rust resistance genes *Sr24* and *Sr26* in diverse wheat germplasm. Theor. Appl. Genet. 111 (3), 496–504. 10.1007/s00122-005-2039-z 15918008

[B115] Mago R. Brown-Guedira G. Dreisigacker S. Breen J. Jin Y. Singh R. (2011). An accurate DNA marker assay for stem rust resistance gene *Sr2* in wheat. Theor. Appl. Genet. 122 (4), 735–744. 10.1007/s00122-010-1482-7 21060985

[B116] Mago R. Zhang P. Vautrin S. Simkova H. Bansal U. Luo M. C. (2015). The wheat *Sr50* gene reveals rich diversity at a cereal disease resistance locus. Nat. Plants 1, 15186. 10.1038/nplants.2015.186 27251721

[B117] Mago R. Till B. Periyannan S. Yu G. Wulff B. B. H. Lagudah E. (2017). “Generation of loss-of-function mutants for wheat rust disease resistance gene cloning,” in Wheat rust diseases: methods and protocols. Ed. Periyannan S. (New York: Springer), 199–205. 10.1007/978-1-4939-7249-4_17 28856652

[B118] Marchal C. Zhang J. Zhang P. Fenwick P. Steuernagel B. Adamski N. M. (2018). BED-domain-containing immune receptors confer diverse resistance spectra to yellow rust. Nat. Plants 4 (9), 662–668. 10.1101/299651 30150615

[B119] Marcussen T. Sandve S. R. Heier L. Spannagl M. Pfeifer M. International Wheat Genome Sequencing C. (2014). Ancient hybridizations among the ancestral genomes of bread wheat. Science 345 (6194):1250092. 10.1126/science.1250092 25035499

[B120] Mayer K. F. Rogers J. Doležel J. Pozniak C. Eversole K. Feuillet C. (2014). A chromosome-based draft sequence of the hexaploid bread wheat (*Triticum aestivum*) genome. Science 345, 6194. 10.1126/science.1251788 25035500

[B121] McFadden E. Sears E. (1946). The origin of *Triticum spelta* and its free-threshing hexaploid relatives. J. Hered. 37, 81–89. 10.1093/oxfordjournals.jhered.a105590 20985728

[B122] McFadden E. S. (1930). Successful transfer of emmer characteristics to vulgare wheat. J. Amer. Soc Agron. 22, 1020–1034. 10.2134/agronj1930.00021962002200120005x

[B123] McIntosh R. A. Watson I. A. (1982). “Genetics of host-pathogen interactions in rusts,” in The rust fungi. Eds. Scott K. J. Chakravorty A. K. (New York: Academic Press), 121–149.

[B124] McIntosh R. A. Wellings C. R. Park R. F. (1995). Wheat rusts: an atlas of resistance genes (Australia: CSIRO), 1–204, ISBN: ISBN 0643054286.

[B125] McIntosh R. A. Bariana H. S. Park R. F. Wellings C. R. (2001). Aspects of wheat rust research in Australia. Euphytica 119, 115–120. 10.1023/A:1017546506246

[B126] McIntosh R. A. Dubcovsky J. Rogers W. J. Morris C. Xia X. C. (2017). Catalogue of gene symbols for wheat: 2017 supplement. (Yokohama, Japan Komugi Wheat Genetic Resources Database). Available at: https://shigen.nig.ac.jp/wheat/komugi/genes/symbolClassList.jsp.

[B127] Michelmore R. W. Paran I. Kesseli R. V. (1991). Identification of markers linked to disease-resistance genes by bulked segregant analysis: a rapid method to detect markers in specific genomic regions by using segregating populations. Proc. Natl. Acad. Sci. 88 (21), 9828–9832. 10.1073/pnas.88.21.9828 1682921 PMC52814

[B128] Milne R. J. Dibley K. E. Schnippenkoetter W. Mascher M. Lui A. C. W. Wang L. (2019). The Wheat *Lr67* Gene from the Sugar Transport Protein 13 Family Confers Multipathogen Resistance in Barley. Plant Physiol. 179 (4), 1285–1297. 10.1104/pp.18.00945 30305371 PMC6446772

[B129] Mohler V. Singh D. Singrün C. Park R. F. (2012). Characterization and mapping of *Lr65 in* spelt wheat ‘Altgold Rotkorn’. Plant Breed. 131 (2), 252–257. 10.1111/j.1439-0523.2011.01934.x

[B130] Money N. P. (2016). “Fungal Diversity,” in The Fungi (Third edition). Ed. Watkinson S. C. Boddy L. Money N. P. (USA: Academic Press), 1–36. 10.1016/B978-0-12-382034-1.00001-3

[B131] Moore J. W. Herrera-Foessel S. Lan C. Schnippenkoetter W. Ayliffe M. Huerta-Espino J. (2015). A recently evolved hexose transporter variant confers resistance to multiple pathogens in wheat. Nat. Genet. 47 (12), 1494–1498. 10.1038/ng.3439 26551671

[B132] Narang D. Kaur S. Steuernagel B. Ghosh S. Bansal U. Li J. (2020). Discovery and characterisation of a new leaf rust resistance gene introgressed in wheat from wild wheat *Aegilops peregrina* . Sci. Rep. 10, 7573. 10.1038/s41598-020-64166-2 32371881 PMC7200655

[B133] Nayar S. K. Bhardwaj S. C. Prashar M. (1999). Characterization of *Lr34* and *Sr2 in* Indian wheat (*Triticum aestivum*) germplasm. Indian J. Agric. Sci. 69, 718–721.

[B134] Nayar S. K. Nagarajan S. Prashar M. Bhardwaj S. C. Jain S. K. Datta D. (2001). Revised catalogue of genes that accord resistance to Puccinia species in wheat. (Flowerdale, Shimla: Directorate of Wheat Research, Regional Station), 1–48.

[B135] Nevo E. Korol A. B. Beiles A. Tzion F. (2013). “Evolution of wild emmer and wheat improvement: population genetics, genetic resources, and genome organization of wheat’s progenitor,” in Triticum dicoccoides (Berlin: Springer Science and Business Media).

[B136] Nsabiyera V. Bariana H. S. Qureshi N. Wong D. Hayden M. J. Bansal U. K. (2018). Characterisation and mapping of adult plant stripe rust resistance in wheat accession Aus27284. Theor. Appl. Genet. 131 (7), 1459–67. 10.1007/s00122-018-3090-x 29560515

[B137] Nsabiyera V. Baranwal D. Qureshi N. Kay P. Forrest K. Valárik M. (2020). Fine mapping of Lr49 using 90K SNP chip array and flow sorted chromosome sequencing in wheat. Front. Plant Sci. 10:1787:1787. 10.3389/fpls.2019.01787 32117347 PMC7010802

[B138] Pakeerathan K. Bariana H. Qureshi N. Wong D. Hayden M. Bansal U. (2019). Identification of a new source of stripe rust resistance Yr82 in wheat. Theor. Appl. Genet. 132, 3169–3176. 10.1007/s00122-019-03416-y 31463519

[B139] Pal D. Bhardwaj S. C. Khan H. Patial M. (2018). HS628: A potential genetic stock for resistance to new virulent pathotypes of black, brown and yellow rusts of wheat (*Triticum aestivum* L.). Wheat Barley Res. 10, 61–63. 10.25174/2249-4065/2018/76849

[B140] Pardey P. G. Beddow J. M. Kriticos D. J. Hurley T. M. Park R. F. Duveiller E. (2013). Right-sizing stem-rust research. Science 340, 147–148. 10.1126/science.122970 23580514

[B141] Park R. Mohler V. Nazari K. Singh D. (2014). Characterisation and mapping of gene *Lr73* conferring seedling resistance to *Puccinia triticina* in common wheat. Theor. Appl. Genet. 127 (9), 2041–2049. 10.1007/s00122-014-2359-y 25116148

[B142] Periyannan S. Moore J. Ayliffe M. Bansal U. Wang X. Huang L. (2013). The gene *Sr33*, an ortholog of barley Mla genes, encodes resistance to wheat stem rust race *Ug99* . Science 341 (6147), 786–788. 10.1126/science.1239028 23811228

[B143] Periyannan S. (2018). Sustaining global agriculture through rapid detection and deployment of genetic resistance to deadly crop diseases. New Pathol. 219, 45–51. 10.1111/nph.14928 29205390

[B144] Person C. O. (1959). Gene-for-gene relationships in host parasite systems. Can. J. Bot. 37 (1), 101–130. 10.1139/b59-087

[B145] Pink D. Hand P. (2002). Plant resistance and strategies for breeding resistant varieties. Plant Protection Sci. 38 (1), 9–14. 10.17221/10310-PPS

[B146] Poland J. A. Rife T. W. (2012). Genotyping-by-sequencing for plant breeding and genetics. Plant Genome 5 (3), 92–102. 10.3835/plantgenome2012.05.0005

[B147] Poland J. A. Brown P. J. Sorrells M. E. Jannink J. L. (2012). Development of high-density genetic maps for barley and wheat using a novel two-enzyme genotyping-by-sequencing approach. PloS One 7, 1–8. 10.1371/journal.pone.0032253 PMC3289635 22389690

[B148] Pont C. Salse J. (2017). Wheat paleohistory created asymmetrical genomic evolution. Curr. Opin. Plant Biol. 36, 29–37. 10.1016/j.pbi.2017.01.001 28182971

[B149] Preece C. Livarda A. Christin P. A. Wallace M. Martin G. Charles M. (2017). How did the domestication of Fertile Crescent grain crops increase their yields? Funct. Ecol. 31 (2), 387–397. 10.1111/1365-2435.12760 28286354 PMC5324541

[B150] Pretorius Z. A. Singh R. P. Wagoire W. W. Payne T. S. (2000). Detection of virulence to wheat stem rust resistance gene Sr31 in *Puccinia graminis.* f. sp. *tritici* in Uganda. Plant Dis. 84, 203–203. 10.1094/PDIS.2000.84.2.203B 30841334

[B151] Qi L. Pumphrey M. Friebe B. Zhang P. Qian C. Bowden R. (2011). A novel Robertsonian translocation event leads to transfer of a stem rust resistance gene (*Sr52*) effective against race *Ug99* from *Dasypyrum villosum* into bread wheat. Theor. Appl. Genet. 123 (1), 159–167. 10.1007/s00122-011-1574-z 21437597

[B152] Qureshi N. Bariana H. Forrest K. Hayden M. Keller B. Wicker T. (2016). Fine mapping of the chromosome 5B region carrying closely linked rust resistance genes *Yr47* and *Lr52 in* wheat. Theor. Appl. Genet. 130, 495–504. 10.1007/s00122-016-2829-5 27866228

[B153] Qureshi N. Bariana H. Zhang P. McIntosh R. Bansal U. Wong D. (2018a). Genetic relationship of stripe rust resistance genes *Yr34* and *Yr48* in wheat and identification of linked KASP markers. Plant Dis. 102 (2), 413–420. 10.1094/PDIS-08-17-1144-RE 30673523

[B154] Qureshi N. Kandiah P. Gessese M. K. Nsabiyera V. Wells V. Babu P. (2018b). Development of co-dominant KASP markers co-segregating with Ug99 effective stem rust resistance gene *Sr26* in wheat. Mol. Breed. 38 (8), 97. 10.1007/s11032-018-0854-6

[B155] Qureshi N. Bariana H. Kumran V. V. Muruga S. Forrest K. L. Hayden M. J. (2018c). A new leaf rust resistance gene *Lr79* mapped in chromosome 3BL from the durum wheat landrace Aus26582. Theor. Appl. Genet. 131 (5), 1091–1098. 10.1007/s00122-018-3060-3 29396589

[B156] Ramirez-Gonzalez R. H. Uauy C. Caccamo M. (2015). PolyMarker: a fast polyploid primer design pipeline. Bioinformatics 31 (12), 2038–2039. 10.1093/bioinformatics/btv069 25649618 PMC4765872

[B157] Randhawa M. Bansal U. Valarik M. Klocova B. Dolezel J. Bariana H. (2014). Molecular mapping of stripe rust resistance gene *Yr51* in chromosome 4AL of wheat. Theor. Appl. Genet. 127 (2), 317–324. 10.1007/s00122-013-2220-8 24185819

[B158] Randhawa M. S. Bains N. S. Sohu V. S. Chhuneja P. Trethowan R. M. Bariana H. S. (2019). Marker Assisted Transfer of Stripe Rust and Stem Rust Resistance Genes into Four Wheat Cultivars. Agronomy 9 (9), 1–10. 10.3390/agronomy9090497

[B159] Ren R. S. Wang M. N. Chen X. M. Zhang Z. J. (2012). Characterization and molecular mapping of *Yr52* for high-temperature adult-plant resistance to stripe rust in spring wheat germplasm PI 183527. Theor. Appl. Genet. 125 (5), 847–857. 10.1007/s00122-012-1877-8 22562146

[B160] Rinaldo A. Gilbert B. Boni R. Krattinger S. G. Singh D. Park R. F. (2017). The *Lr34* adult plant rust resistance gene provides seedling resistance in durum wheat without senescence. Plant Biotechnol. J. 15 (7), 894–905. 10.1111/pbi.12684 28005310 PMC5466443

[B161] Risk J. M. Selter L. L. Chauhan H. Krattinger S. G. Kumlehn J. Hensel G. (2013). The wheat *Lr34* gene provides resistance against multiple fungal pathogens in barley. Plant Biotechnol. J. 11 (7), 847–854. 10.1111/pbi.12077 23711079

[B162] Röder M. S. Korzun V. Wendehake K. Plaschke J. Tixier M. H. Leroy P. (1998). A microsatellite map of wheat. Genetics 149 (4), 2007–2023. 9691054 10.1093/genetics/149.4.2007 PMC1460256

[B163] Roelfs A. P. Singh R. P. Saari E. E. (1992). Rust diseases of wheat: concepts and methods of disease management (Mexico: CIMMYT).

[B164] Roelfs A. P. (1978). Estimated losses caused by rust in small grains cereals in the United States (United States: United States Department of Agriculture: Agricultural Research Service. 89), 1918–1976.

[B165] Roelfs A. P. (1985). Wheat and Rye Stem Rust, in The cereal rusts: diseases, distribution, epidemiology, and control. Eds. Roelfs A. P. Bushnell W. R. (Orlando, FL, USA: Academic Press), 3–37.

[B166] Ronen G. Carmel-Goren L. Zamir D. Hirschberg J. (2000). An alternative pathway to beta -carotene formation in plant chromoplasts discovered by map-based cloning of beta and old-gold color mutations in tomato. Proc. Natl. Acad. Sci. U. S. A. 97 (20), 11102–11107. 10.1073/pnas.190177497 10995464 PMC27155

[B167] Rosewarne G. M. Singh R. P. Huerta-Espino J. Herrera-Foessel S. A. Forrest K. L. Hayden M. J. (2012). Analysis of leaf and stripe rust severities reveals pathotype changes and multiple minor QTLs associated with resistance in an Avocet × Pastor wheat population. Theor. Appl. Genet. 124 (7), 1283–1294. 10.1007/s00122-012-1786-x 22274764

[B168] Rosewarne G. Herrera-Foessel S. Singh R. Huerta-Espino J. Lan C. He Z. (2013). Quantitative trait loci of stripe rust resistance in wheat. Theor. Appl. Genet. 126 (10), 2427–2449. 10.1007/s00122-013-2159-9 23955314 PMC3782644

[B169] Rowell J. B. Roelfs A. P. (1976). “Wheat stem rust In Modeling for Pest Management, Concepts, Techniques, and Applications U.S.A./U.S.S.R,” in 2nd U.S./U.S.S.R. Symp. Eds. Tummala R. L. Haynes D. L. Croft B. A. (East Lansing: Michigan State University), 69–79.

[B170] Russel G. E. (1978). Plant breeding for pest and disease resistance (London: Butterworth and Co. (Publisher) Ltd), pp 16–pp 25.

[B171] Saini R. G. Kaur M. Singh B. Sharma S. Nanda G. S. Nayar S. K. (2002). Genes *Lr48* and *Lr49* for hypersensitive adult plant leaf rust resistance in wheat (*Triticum aestivum* L.). Euphytica 124 (3), 365–370. 10.1023/a:1015762812907

[B172] Saintenac C. Jiang D. Wang S. Akhunov E. (2013a). Sequence-based mapping of the polyploid wheat genome. G3 (Bethesda) 3 (7), 1105–1114. 10.1534/g3.113.005819 23665877 PMC3704239

[B173] Saintenac C. Zhang W. Salcedo A. Rouse M. N. Trick H. N. Akhunov E. (2013b). Identification of wheat gene *Sr35* that confers resistance to Ug99 stem rust race group. Science 341, 783–786. 10.1126/science.1239022 23811222 PMC4748951

[B174] Sanchez-Martin J. Steuernagel B. Ghosh S. Herren G. Hurni S. Adamski N. (2016). Rapid gene isolation in barley and wheat by mutant chromosome sequencing. Genome Biol. 17, 221. 10.1186/s13059-016-1082-1 27795210 PMC5087116

[B175] Sandve S. R. Marcussen T. Mayer K. Jakobsen K. S. Heier L. Steuernagel B. (2015). Chloroplast phylogeny of Triticum/Aegilops species is not incongruent with an ancient homoploid hybrid origin of the ancestor of the bread wheat D-genome. New Phytol. 208, 9–10. 10.1111/nph.13487 25996653

[B176] Sawhney R. N. Sharma D. V. (1990). Identification of sources for rust resistance and for durability to Leaf rust in common wheat. SABRAO J. 22, 51–55.

[B177] Sawhney R. N. Goel L. B. Mathur H. C. (1982). Adult plant responses of specific genes resistant to leaf rust against Indian population of leaf rust pathogen. Z. Pflanzenzuchtg 89, 222–228.

[B178] Schnippenkoetter W. Lo C. Liu G. Dibley K. Chan W. L. White J. (2017). The wheat *Lr34* multipathogen resistance gene confers resistance to anthracnose and rust in sorghum. Plant Biotechnol. J. 15 (11), 1387–1396. 10.1111/pbi.12723 28301718 PMC5633760

[B179] Schwessinger B. (2017). Fundamental wheat stripe rust research in the 21(st) century. New Phytol. 213, 1625–1631. 10.1111/nph.14159 27575735

[B180] Shank R. (1994). “Wheat stem rust and drought effects on bale agricultural production and future prospects. Report on February 17–28 assessment,” in In United Nations Emergencies Unit for Ethiopia (Ethiopia: Addis Ababa).

[B181] Shewry P. R. (2009). Wheat. J. Exp. Bot. 60 (6), 1537–1553. 10.1093/jxb/erp058 19386614

[B182] Singh R. P. Rajaram S. (1993). Genetics of adult plant resistance to stripe rust in ten spring bread wheats. Euphytica 72, 1–7. 10.1007/BF00023766

[B183] Singh R. P. Trethowan R. (2007). “Breeding spring bread wheat for irrigated and rainfed production system of developing world,” in a book “Breeding major food staples”, vol. pp . Eds. Kang M. Priyadarshan P. M. (Iowa, USA: Blackwell Publishing), 109–140. 10.1002/9780470376447.ch5

[B184] Singh R. P. Hong M. Huerta-Espino J. (1994). “Rust diseases of wheat. In a book “Guide to the CIMMYT Wheat Crop Protection Sub-Program”,” in Wheat special report number 24. Eds. Saari E. E. Hettel G. P. (Mexico: CIMMYT), 19–33.

[B185] Singh R. P. Huerta-Espino J. Rajaram S. (2000). Achieving near-immunity to leaf and stripe rusts in wheat by combining slow rusting resistance genes. Acta Phytopathol. Entomol. Hung. 35 (1/4), 133–139.

[B186] Singh D. Park R. F. McIntosh R. A. (2001). Postulation of leaf (brown) rust resistance genes in 70 wheat cultivars grown in the United Kingdom. Euphytica 120, 205–218. 10.1023/A:1017578217829

[B187] Singh R. P. Rajaram S. Saini R. G. Huerta-Espino J. William M. (2004a). “Genetics of host-pathogen interaction and breeding for durable resistance,” in In a book “Plant Breeding- Mendelian to molecular approaches”. Eds. Jain H. K. Kharkwal M. C. (New Delhi, India: Narosa Publishing House), 145–166. 10.1007/978-94-007-1040-5_7

[B188] Singh R. P. Huerta-Espino J. Pfeiffer W. Figueroa-Lopez P. (2004b). Occurrence and impact of a new leaf rust race on durum wheat in Northwestern Mexico from 2001 to 2003. Plant Dis. 88 (7), 703–708. 10.1094/PDIS.2004.88.7.703 30812479

[B189] Singh S. S. Sharma R. K. Singh G. Tyagi B. S. Saharan M. S. (2011). 100 Years of wheat research in India- A saga of distinguished achievements. (Karnal, India: Directorate of Wheat Research), 281.

[B190] Singh D. Mohler V. Park R. (2013). Discovery, characterisation and mapping of wheat leaf rust resistance gene *Lr71* . Euphytica 190 (1), 131–136. 10.1007/s10681-012-0786-x

[B191] Singh R. P. Herrera-Foessel S. Huerta-Espino J. Lan C. Basnet B. Bhavani S. (2013). “Pleiotropic gene Lr46/Yr29/Pm39/Ltn2 confers slow rusting, adult plant resistance to wheat stem rust fungus,” in Proceedings Paper presented at the Borlaug Global Rust Initiative (New Delhi India: Technical Workshop).

[B192] Singh R. P. Herrera-Foessel S. Huerta-Espino J. Singh S. Bhawani S. Lan C. (2014). Progress towards Genetics and Breeding for Minor Genes based Resistance to Ug99 and other Rusts in CIMMYT High Yielding Spring Wheat. J. Integr. Agric. 13, 255–261. 10.1016/S2095-3119(13)60649-8

[B193] Singh R. P. Hodson D. P. Jin Y. Lagudah E. S. Ayliffe M. A. Bhavani S. (2015). Emergence and spread of new races of wheat stem rust fungus: continued threat to food security and prospects of genetic control. Phytopathology 105 (7), 872–884. 10.1094/PHYTO-01-15-0030-FI 26120730

[B194] Singh R. P. (1992). Genetic association of leaf rust resistance gene *Lr34* with adult plant resistance to stripe rust in bread wheat. Phytopath 82, 835–838. 10.1094/Phyto-82-835

[B195] Singla J. Lüthi L. Wicker T. Bansal U. Krattinger S. G. Keller B. (2017). Characterization of *Lr75*: a partial, broad-spectrum leaf rust resistance gene in wheat. Theor. Appl. Genet. 130, 1–12. 10.1007/s00122-016-2784-1 27659842

[B196] Slinkard A. E. McIntosh R. A. (1998). “Proceedings of the 9th international wheat genetics symposium,” in International wheat genetics symposium (University of Saskatchewan: University Extension Press).

[B197] Sobhy Draz I. (2019). Common Ancestry of Egyptian *Puccinia striiformis* Population Along with Effective and Ineffective Resistance Genes. Asian J. Biol. Sci. 12 (2), 217–221. 10.3923/ajbs.2019.217.221

[B198] Somers D. J. Isaac P. Edwards K. (2004). A high-density microsatellite consensus map for bread wheat (*Triticum aestivum* L.). Theor. Appl. Genet. 109, 1105–1114. 10.1007/s00122-0041740-7 15490101

[B199] Song Q. Shi J. Singh S. Fickus E. Costa J. Lewis J. (2005). Development and mapping of microsatellite (SSR) markers in wheat. Theor. Appl. Genet. 110 (3), 550–560. 10.1007/s00122-004-1871-x 15655666

[B200] Sørensen C. K. Hovmøller M. S. Leconte M. Dedryver F. de Vallavieille-Pope C. (2014). New races of *Puccinia striiformis* found in Europe reveal race specificity of long-term effective adult plant resistance in wheat. Phytopathology 104 (10), 1042–1051. 10.1094/PHYTO-12-13-0337-R 24624957

[B201] Steuernagel B. Periyannan S. K. Hernandez-Pinzon I. Witek K. Rouse M. N. Yu G. (2016). Rapid cloning of disease-resistance genes in plants using mutagenesis and sequence capture. Nat. Biotechnol. 34 (6), 652–655. 10.1038/nbt.3543 27111722

[B202] Sthapit J. Gbur E. E. Brown-Guedira G. Marshall D. S. Milus E. A. (2012). Characterization of resistance to stripe rust in contemporary cultivars and lines of winter wheat from the eastern United States. Plant Dis. 96 (5), 737–745. 10.1094/PDIS-07-11-0612 30727527

[B203] Stoa T. E. (1945). A brief history of wheat variety changes on farms in North Dacota. Bi-monthly Bull. 7 (6), 21–26.

[B204] Tang Y. Liu X. Wang J. Li M. Wang Q. Tian F. (2016). GAPIT version 2: an enhanced integrated tool for genomic association and prediction. Plant Genome 9 (2), 1–9. 10.3835/plantgenome2015.11.0120 27898829

[B205] Tavares S. Ramos A. P. Pires A. S. Azinheira H. G. Caldeirinha P. Link T. (2014). Genome size analyses of Pucciniales reveal the largest fungal genomes. Front. Plant Sci. 5, 422. 10.3389/fpls.2014.00422 25206357 PMC4143883

[B206] Thind A. Wicker T. Šimková H. Fossati D. Moullet O. Brabant C. (2017). Rapid cloning of genes in hexaploid wheat using cultivar-specific long-range chromosome assembly. Nat. Biotechnol. 35, 793–796. 10.1038/nbt.3877 28504667

[B207] Van der Plank J. E. (1963). Plant diseases: epidemics and control. Acad. Press New Y. p, 349.

[B208] Vrána J. Šimková H. Kubaláková M. Číhalíková J. Doležel J. (2012). Flow cytometric chromosome sorting in plants: the next generation. Methods 57, 331–337. 10.1016/j.ymeth.2012.03.006 22440520

[B209] Walter S. Ali S. Kemen E. Nazari K. Bahri B. A. Enjalbert J. (2016). Molecular markers for tracking the origin and worldwide distribution of invasive strains of *Puccinia striiformis* . Ecol. Evol. 6 2790, 2804. 10.1002/ece3.2069 PMC4800029 27066253

[B210] Wang D. G. Fan J. B. Siao C. J. Berno A. Young P. Sapolsky R. (1998). Large-scale identification, mapping, and genotyping of single-nucleotide polymorphisms in the human genome. Science 280 (5366), 1077–1082. 10.1126/science.280.5366.1077 9582121

[B211] Wang S. Wong D. Forrest K. Allen A. Chao S. Huang B. E. (2014). Characterization of polyploid wheat genomic diversity using a high-density 90000 single nucleotide polymorphism array. Plant Biotechnol. J. 12 (6), 787–796. 10.1111/pbi.12183 24646323 PMC4265271

[B212] Wang M. N. Wan A. M. Chen X. M. (2015). Barberry as alternate host is important for *Puccinia graminis* f. sp. *tritici* but not for *Puccinia striiformis* f. sp. *tritici* in the U.S. Pacific Northwest. Plant Dis. 99, 1507–1516. 10.1094/PDIS-12-14-1279-RE 30695965

[B213] Wang Z. Li J. Chen S. Heng Y. Chen Z. Yang J. (2017). Poaceae specific *MS1* encodes a phospholipid-binding protein for male fertility in bread wheat. Proc. Natl. Acad. Sci. U. S. A. 114, 12614–12619. 10.1073/pnas.1715570114 29109252 PMC5703327

[B214] Wang K. Riaz B. Ye X. (2018). Wheat genome editing expedited by efficient transformation techniques: progress and perspectives. Crop J. 6 (1), 22–31. 10.1016/j.cj.2017.09.009

[B215] Watanabe S. Hideshima R. Xia Z. Tsubokura Y. Sato S. Nakamoto Y. (2009). Map-based cloning of the gene associated with the soybean maturity locus E3. Genetics 182 (4), 1251–1262. 10.1534/genetics.108.098772 19474204 PMC2728863

[B216] Watson I. A. (1981). “Wheat and its rust parasites in Australia,” in In a book “Wheat Science Today and Tomorrow”, Eds. Evans L. T. Peacock W. J. (Cambridge: Cambridge University Press), 129–147.

[B217] Wellings C. R. (2011). Global status of stripe rust: a review of historical and current threats. Euphytica 179 (1), 129–141. 10.1007/s10681-011-0360-y

[B218] Wessels E. Botes W. C. (2014). Accelerating resistance breeding in wheat by integrating marker-assisted selection and doubled haploid technology. S. Afr. J. Plant Soil 31 (1), 35–43. 10.1080/02571862.2014.903434

[B219] Wilcoxon R. D. (1981). Genetics of Slow Rusting in Cereals. Phytopathology 71, 989–993. 10.1094/Phyto-71-989

[B220] William M. Singh R. P. Huerta-Espino J. Ortiz Islas S. Hoisington D. (2003). Molecular marker mapping of leaf rust resistance gene *Lr46* and its association with stripe rust resistance gene *Yr29 in* Wheat. Phytopathology 93 (2), 153–159. 10.1094/PHYTO.2003.93.2.153 18943129

[B221] Wu J. Wang Q. Xu L. Chen X. Li B. Mu J. (2018). Combining SNP genotyping array with bulked segregant analysis to map a gene controlling adult-plant resistance to stripe rust in wheat line 03031-1-5 H62. Phytopathology 108 (1), 103–113. 10.1094/PHYTO-04-17-0153-R 28832276

[B222] Xu L. S. Wang M. N. Cheng P. Kang Z. Hulbert S. Chen X. (2013). Molecular mapping of *Yr53*, a new gene for stripe rust resistance in durum wheat accession PI 480148 and its transfer to common wheat. Theor. Appl. Genet. 126 (2), 523–533. 10.1007/s00122-012-1998-0 23090143

[B223] Yang H. Tao Y. Zheng Z. Li C. Sweetingham M. W. Howieson J. G. (2012). Application of next-generation sequencing for rapid marker development in molecular plant breeding: a case study on anthracnose disease resistance in *Lupinus angustifolius* L. BMC Genomics 13 1–12. 10.1186/1471-2164-13-318 22805587 PMC3430595

[B224] Yang E. N. Rosewarne G. M. Herrera-Foessel S. A. Huerta-Espino J. Tang Z. X. Sun C. F. (2013). QTL analysis of the spring wheat “Chapio” identifies stable stripe rust resistance despite inter-continental genotype × environment interactions. Theor. Appl. Genet. 126 (7), 1721–1732. 10.1007/s00122-013-2087-8 23558982

[B225] Yao F. Zhang X. Ye X. Li J. Long L. Yu C. (2019). Characterization of molecular diversity and genome-wide association study of stripe rust resistance at the adult plant stage in Northern Chinese wheat landraces. BMC Genet. 20, 38. 10.1186/s12863-019-0736-x 30914040 PMC6434810

[B226] Yin J. L. Fang Z. W. Sun C. Zhang P. Zhang X. Lu C. (2018). Rapid identification of a stripe rust resistant gene in a space-induced wheat mutant using specific locus amplified fragment (SLAF) sequencing. Sci. Rep. 8, 3086. 10.1038/s41598-018-21489-5 29449594 PMC5814476

[B227] Yu J. Buckler E. S. (2006). Genetic association mapping and genome organization of maize. Curr. Opin. Biotechnol. 17 (2), 155–160. 10.1016/j.copbio.2006.02.003 16504497

[B228] Yu L. X. Lorenz A. Rutkoski J. Singh R. P. Bhavani S. Huerta-Espino J. (2011). Association mapping and gene–gene interaction for stem rust resistance in CIMMYT spring wheat germplasm. Theor. Appl. Genet. 3 (8), 1257–1268. 10.1007/s00122-011-1664-y 21811818

[B229] Yu L. X. Barbier H. Rouse M. N. Singh S. Singh R. P. Bhavani S. (2014). A consensus map for *Ug99* stem rust resistance loci in wheat. Theor. Appl. Genet. 127 (7), 1561–1581. 10.1007/s00122-014-2326-7 24903979 PMC4072096

[B230] Zadoks J. C. Bouwman J. J. (1985). “Epidemiology in Europe,” in The Cereal Rusts, vol. Vol. 2 . Eds. Roelfs A. P. Bushnell W. R. (Orlando, FL: Academic Press), 329–369.

[B231] Zadoks J. C. (1985). On the conceptual basis of crop loss assessment: the threshold theory. A. Rev. Phytopathol. 23, 455–473. 10.1146/annurev.py.23.090185.002323

[B232] Zhang J. Lu Y. Yuan Y. Zhang X. Geng J. Chen Y. (2009). Map-based cloning and characterization of a gene controlling hairiness and seed coat color traits in Brassica rapa. Plant Mol. Biol. 69 (5), 553–563. 10.1007/s11103-008-9437-y 19039665

[B233] Zhang Z. Ersoz E. Lai C. Q. Todhunter R. J. Tiwari H. K. Gore M. A. (2010). Mixed linear model approach adapted for genome-wide association studies. Nat. Gen. 42 (4), 355. 10.1038/ng.546 PMC2931336 20208535

[B234] Zhang D. Bowden R. L. Yu J. Carver B. F. Bai G. (2014). Association analysis of stem rust resistance in US winter wheat. PloS One 9 (7), e103747. 10.1371/journal.pone.0103747 25072699 PMC4114971

[B235] Zhang W. Chen S. Abate Z. Nirmala J. Rouse M. N. Dubcovsky J. (2017). Identification and characterization of *Sr13*, a tetraploid wheat gene that confers resistance to the *Ug99* stem rust race group. Proc. Natl. Acad. Sci. U. S. A. 114 (45), E9483–E9492. 10.1073/pnas.1706277114 29078294 PMC5692537

[B236] Zhang C. Huang L. Zhang H. Hao Q. Lyu B. Wang M. (2019). An ancestral NB-LRR with duplicated 3′ UTRs confers stripe rust resistance in wheat and barley. Nat. Commun. 10, 1–12. 10.1038/s41467-019-11872-9 31492844 PMC6731223

[B237] Zhang J. Zhang P. Dodds P. Lagudah E. (2020). How Target-Sequence Enrichment and Sequencing (TEnSeq) Pipelines Have Catalyzed Resistance Gene Cloning in the Wheat-Rust Pathosystem. Front. Plant Sci. 11, 678. 10.3389/fpls.2020.00678 32528511 PMC7264398

[B238] Zhou X. Wang M. Chen X. Lu Y. Kang Z. Jing J. (2014). Identification of *Yr59* conferring high-temperature adult-plant resistance to stripe rust in wheat germplasm PI 178759. Theor. Appl. Genet. 127 (4), 935–945. 10.1007/s00122-014-2269-z 24487945

[B239] Zhou X. L. Han D. J. Chen X. M. Gou H. L. Guo S. J. Rong L. (2014a). Characterization and molecular mapping of stripe rust resistance gene *Yr61* in winter wheat cultivar Pindong 34. Theor. Appl. Genet. 127 (11), 2349–2358. 10.1007/s00122-014-2381-0 25163935

